# Tau Pathology Drives Disease‐Associated Astrocyte Reactivity in Salt‐Induced Neurodegeneration

**DOI:** 10.1002/advs.202410799

**Published:** 2025-01-24

**Authors:** Tong‐Yu Rui, He‐Zhou Huang, Kai Zheng, Hong‐Wei Fan, Juan Zhang, Zi‐Yuan Guo, Heng‐Ye Man, Nadezhda Brazhe, Alexey Semyanov, You‐Ming Lu, Dan Liu, Ling‐Qiang Zhu

**Affiliations:** ^1^ Department of Pathophysiology School of Basic Medicine Tongji Medical College Huazhong University of Science and Technology Wuhan Hubei 430030 China; ^2^ Department of Geriatrics Tongji Hospital Tongji Medical College Huazhong University of Science and Technology Wuhan 430030 China; ^3^ Center for Stem Cell and Organoid Medicine (CuSTOM) Division of Developmental Biology Cincinnati Children's Hospital Medical Center Cincinnati OH 45229 USA; ^4^ Department of Biology Boston University Boston MA 02215 USA; ^5^ Faculty of Biology M.V. Lomonosov Moscow State University Moscow 119234 Russia; ^6^ College of Medicine Jiaxing University Jiaxing Zhejiang Province 314001 China; ^7^ Department of Medical Genetics School of Basic Medicine Tongji Medical College Huazhong University of Science and Technology Wuhan Hubei 430030 China

**Keywords:** A1R, Alzheimer's disease, disease‐associated astrocyte, high salt diet, tau pathology

## Abstract

Dietary high salt intake is increasingly recognized as a risk factor for cognitive decline and dementia, including Alzheimer’s disease (AD). Recent studies have identified a population of disease‐associated astrocytes (DAA)‐like astrocytes closely linked to amyloid deposition and tau pathology in an AD mouse model. However, the presence and role of these astrocytes in high‐salt diet (HSD) models remain unexplored. In this study, it is demonstrated that HSD significantly induces enhanced reactivity of DAA‐like astrocytes in the hippocampal CA3 region of mice, with this reactivity being critically dependent on neuronal tau pathology. Neuronal tau pathology activates adenosine A1R signaling, exacerbating tau pathology by inhibiting the Cers1 pathway, which sustains astrocyte reactivity. Additionally, neurons burdened with tau pathology promote astrocyte reactivity via releasing Proteins Associated with Promoting DAA‐like Astrocyte Reactivity (PAPD), with Lcn2 playing a pivotal role. Knockout of Lcn2 or its receptor 24p3R significantly mitigates HSD‐induced DAA reactivity and neuroinflammation. These findings suggest a vicious cycle between tau pathology and A1R signaling, driving DAA‐like astrocyte reactivity. Targeting the Tau‐A1R axis may provide a novel therapeutic strategy for reducing HSD‐induced neuroinflammation and cognitive deficits.

## Graphical abstract

This study reveals that a high‐salt diet (HSD) induces enhanced reactivity of DAA‐like astrocytes in the hippocampal CA3 region via a tau pathology‐A1R signaling axis, forming a vicious cycle that perpetuates astrocyte reactivity. Neuronal tau pathology releases PAPDs, notably Lcn2, driving neuroinflammation. Targeting the Tau‐A1R axis offers therapeutic potential for HSD‐induced cognitive deficits and neurodegeneration.

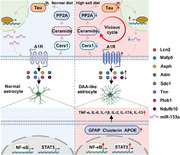

## 1 Introduction

High salt intake has been consistently linked to cerebrovascular diseases and cognitive impairments,^[^ ^1^, ^2^ ^]^ serving as an independent risk factor for both stroke and dementia.^[^ ^2^, ^3^, ^4^ ^]^ Increasing evidence suggests that high‐salt diet (HSD) can promote Alzheimer's disease (AD)‐like pathology. A hallmark of Alzheimer's disease is the accumulation of β‐amyloid (Aβ) plaques and the formation of neurofibrillary tangles.^[^ ^5^, ^6^, ^7^ ^]^ In mice, a high‐salt diet elevates IL‐17 levels, which inhibits eNOS, reducing vascular NO levels. This leads to decreased nitrosylation of neuronal calcium calpain, activating cyclin‐dependent kinase‐5, and ultimately causing tau phosphorylation.^[^ ^8^ ^]^ Additionally, dietary salt disrupts the tricarboxylic acid cycle, leading to excessive tau protein phosphorylation and synaptic dysfunction during aging.^[^ ^9^ ^]^ Thus, the neuronal tau pathology, as seen in the AD brain, is critical to high‐salt diets‐induced memory decline.^[^ ^10^ ^]^

Besides the tau pathology, previous research suggests that neuroinflammation within the central nervous system (CNS) plays a crucial role in the pathological progression of AD. And, similar brain pathological changes, such as glial reactivity, neuroinflammation, and synaptic dysfunction,^[^ ^9^, ^10^, ^11^, ^12^, ^13^, ^14^, ^15^, ^16^, ^17^ ^]^ had been reported in both high‐salt diet and AD brains. In the neuroinflammatory response associated with AD, astrocytes are considered one of the key contributors.^[^ ^18^, ^19^ ^]^ Although reactive astrocytes can engulf pathological tau proteins, their sustained reactivity triggers inflammation‐related signaling pathways within these tau‐engulfing cells, promoting the spread and release of pathological tau and thereby exacerbating neuroinflammation.^[^ ^20^, ^21^ ^]^ Pathological tau proteins further drive neurodegeneration and neuroinflammation by promoting the formation of double‐stranded RNA (dsRNA) derived from retrotransposons.^[^ ^22^ ^]^ Moreover, neuronal tau pathology mediates the interaction between neurons and astrocytes, leading to the proliferation of reactive astrocytes.^[^ ^23^ ^]^ In addition, astrocytic expression of 4R tau drives reactivity and dysfunction in astrocytes, resulting in phenotypes with diminished glutamate uptake capacity and increased sensitivity to oxidative stress.^[^ ^24^ ^]^ The transcriptional state of astrocytes also modulates the progression of tau pathology; for instance, the removal of astrocytic APOE4 has been shown to reduce tau‐induced synaptic loss and microglial phagocytosis of synaptic elements.^[^ ^25^ ^]^ Furthermore, clearing senescent glial cells can prevent tau‐dependent pathology and cognitive decline.^[^ ^26^ ^]^ These lines of evidence suggest that the states of astrocytes are highly correlated with the progression of AD.

Many years ago, the reactive astrocytes could be classified into two broad states: A1 and A2.^[^ ^27^, ^28^ ^]^ However, this broad subtyping hinders the study of specific mechanisms unique to particular diseases, and this classification is no longer recommended.^[^ ^27^ ^]^ Single‐cell transcriptomics and spatial transcriptomics provide a molecular definition of each cell type, capturing their precise morphological and functional characteristics. This approach enables a more nuanced understanding of cellular subpopulation classification, specific spatial localization, and the unique transcriptional features of each subgroup. A state of disease‐associated astrocytes (DAAs) has been identified in 5 × FAD mice and aged human brains using single‐cell transcriptomics, physiological and morphological.^[^ ^29^ ^]^ These DAAs are located adjacent to amyloid plaques in the hippocampus and subiculum and express a unique set of genes, including GFAP, Clusterin, APOE, Serpina3n, and Cathepsin B, which are involved in processes like endocytosis, the complement cascade, and aging. DAAs appear in AD mouse models before cognitive decline and increase as the disease progresses.^[^ ^30^ ^]^ STARmap PLUS allows comprehensive in situ localization of single‐cell transcriptional states and tissue pathology in a mouse model of AD and revealed that a core‐shell structure, with DAA‐like astrocytes primarily enriched within 10–20 µm of the plaques, while phosphorylated tau was mainly enriched at a distance of 10 µm from the plaques.^[^ ^31^ ^]^ However, in the HSD model, which is used to study sporadic AD, whether this DAA‐like astrocyte contributes to the pathogenesis of HSD mice, a sporadic AD model, remains unclear.

Here, we demonstrate that HSD significantly activates DAA‐like astrocytes in the hippocampal CA3 region of mice, with this reactivity being critically dependent on neuronal tau pathology. We reveal that tau pathology and adenosine A1R signaling form a vicious cycle, driving the pathological reactivity of DAA‐like astrocytes. Furthermore, we identify that neurons burdened with tau pathology promote the reactivity of these astrocytes through the release of PAPD, with Lcn2 playing a crucial role. Notably, the knockout of Lcn2 or its receptor 24p3R significantly reduces HSD‐induced DAA reactivity and neuroinflammation.

## 2 Results

### 2.1 High‐Salt Diet Induces Reactivity of DAA‐Like Astrocytes

**Figure 1:**
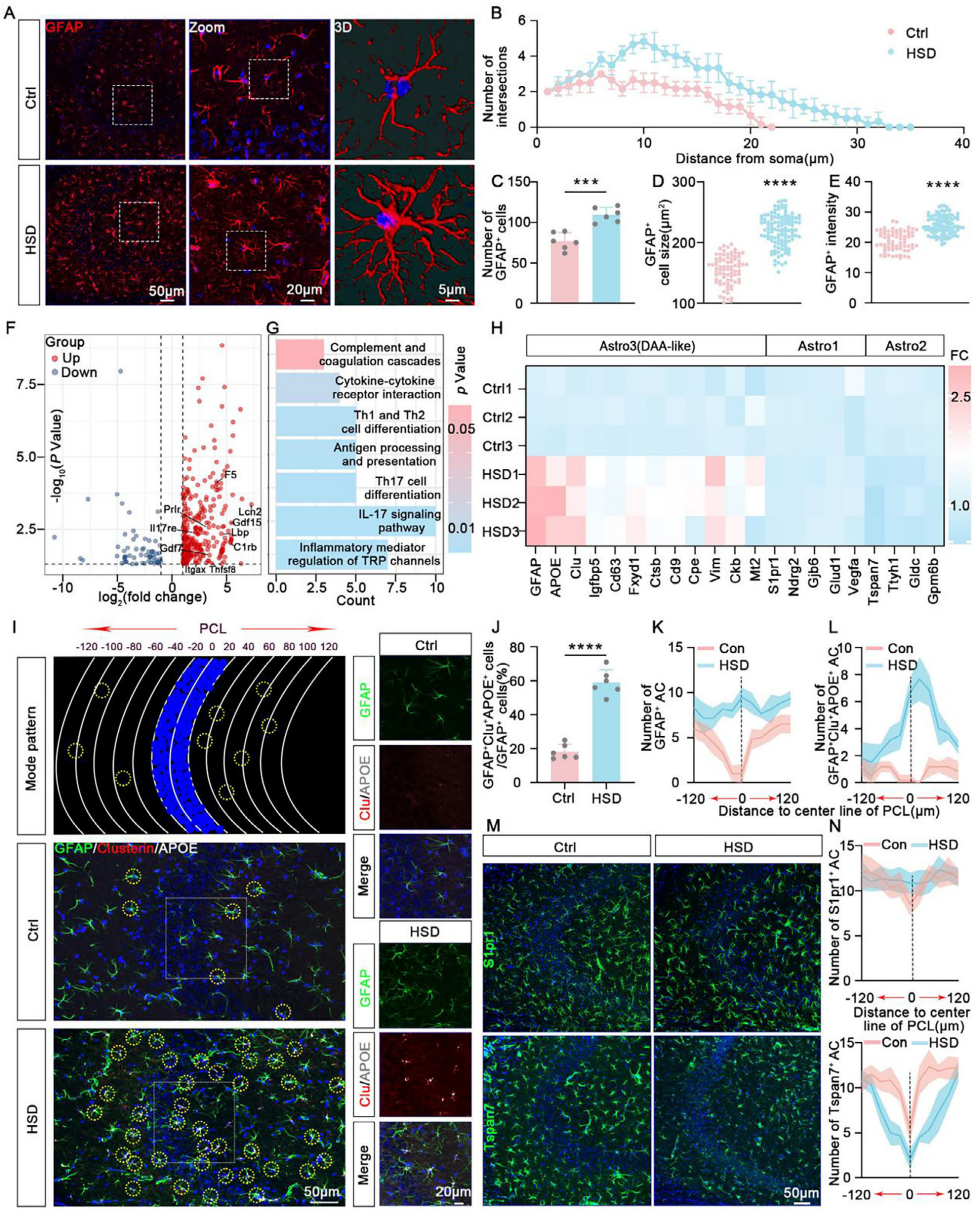
High‐salt diet induces reactivity of DAA‐like astrocytes. A–E) The analysis for the reactive astrocytes in Ctrl (control) mice and HSD. The representative immunofluorescence images for GFAP (red) and 3D reconstruction in the hippocampal CA3 region of Ctrl mice and HSD mice. Blue staining represents DAPI (A). The quantification for the sholl analysis of branches (B), the total number of GFAP^+^ cells (C), the GFAP^+^ cell size (D), and the intensity of GFAP (E). *n* = 6 hippocampal slices for each group. F) Volcano plots showing differential expression of mRNA profiles in the hippocampus of HSD mice compared to Ctrl mice. FC > 1 and p < 0.05 are indicated by the dashed lines. G) GO enrichment analysis of deregulated genes in HSD mice compared with Ctrl mice. H) qPCR to examine the representative genes of Astro1, Astro2, and Astro3 (DAA‐like) in astrocytes sorted by magnetic beads from the hippocampus of Ctrl mice and HSD mice. *n* = 3 mice for each group. I) The diagram to investigate the spatial distribution of DAA‐like astrocytes (upper panel) and the representative immunofluorescence images of DAA‐like astrocytes in the hippocampal CA3 region from Ctrl mice (middle panel) and HSD mice (lower panel). Green: anti‐GFAP, red: anti‐Clusterin, white: anti‐APOE. Blue staining represents DAPI. PCL stands for pyramidal cell layer. A row of curved lines in the upper panel indicates the distance to the pyramidal cell layer and a 20 µm interval is set between lines. The yellow circles indicate the DAA‐like astrocytes. The white rectangles in the middle and lower panels indicate the amplified regions as shown on the right sides with separated images. J) The proportion of GFAP^+^Clu^+^APOE^+^ astrocytes in total GFAP^+^ astrocytes. *n* = 6 hippocampal slices for each group. K,L) The spatial distribution of GFAP^+^ astrocytes (K) and GFAP^+^Clu^+^APOE^+^ astrocytes (L) toward the center of the pyramidal cell layer. *n* = 6 hippocampal slices for each group. M) The representative immunofluorescence images for Astro1 (S1pr1, green) (upper panel) and Astro2 (Tspan7, green) (lower panel) in the hippocampal CA3 region of Ctrl mice and HSD mice. Blue staining represents DAPI. N) The spatial distribution of S1pr1+ astrocytes (upper panel) and Tspan7+ astrocytes (lower panel) toward the center of the pyramidal cell layer. *n* = 6 hippocampal slices for each group.

To investigate the impact of HSD on astrocytes in the brain, we replicated a previously established high‐salt animal model,^[^ ^8^ ^]^ in which animals were fed a custom diet containing 8% salt and water containing 1% salt (Figure S1A, Supporting Information). Consistently, we found that one‐month HSD is sufficient to induce spatial memory impairments in mice, as assessed by novel location recognition, T‐maze, and water maze tests (Figure S1B–I, Supporting Information). In addition, HSD mice also show impaired synaptic transmission and dendritic morphology as shown in mEPSCs, LTP recordings, and Golgi staining (Figure S1J–S, Supporting Information). Upon validating the successful replication of the HSD model, we then employed immunofluorescence and three‐dimensional reconstruction to investigate the morphological changes in astrocytes induced by HSD. We observed that the HSD induced significant astrocyte reactivity, evidenced by enhanced process interactions, increased number of GFAP‐positive reactive astrocytes, enlarged cell size, and elevated GFAP intensity (**Figure** **1**A–E). We also performed RNA‐seq from the hippocampus of HSD and normal diet mice and found that numerous upregulated genes in the HSD mice are significantly associated with neuroinflammation. Pathway enrichment analysis indicated these genes are highly correlated with immune cell differentiation, antigen presentation processes, complement cascades, and cytokine interactions (Figure 1F,G). By employing techniques from previous studies that analyzed astrocyte subpopulations in AD mice,^[^ ^31^ ^]^ we performed an in‐depth analysis of astrocytes from HSD mice after magnetic bead sorting. We found that genes associated with the Astro3 subpopulation (DAA‐like) exhibited significant upregulation in astrocytes from the HSD group, while those linked to Astro2 showed marked downregulation, with no significant change observed in genes related to Astro1 (Figure 1H). To further confirm the DAA‐like reactivity of astrocytes in the mice with an HSD, we performed immunostaining with representative markers of different subtypes. Although we know that DAA‐like astrocytes are indeed a subset of GFAP‐positive astrocytes, relying solely on the GFAP marker is inadequate for labeling DAA‐like astrocytes. Therefore, we opted to co‐label GFAP with two other markers, Clusterin and APOE, which exhibited significant variances in the HSD mice, to ascertain the distribution and proportion characteristics of DAA‐like astrocytes. We found that in the HSD group, the proportion of GFAP^+^Clu^+^APOE^+^ astrocytes (DAA‐like) was significantly upregulated (Figure 1I,J). It is known that reactive astrocytes rarely invade the CA3 pyramidal cell layer of the hippocampus under normal circumstances, however, we observed a significant aggregation of reactive astrocytes (Figure 1K), especially the DAA‐like astrocytes (Figure 1L) toward the pyramidal cell layer in the HSD mice. We also analyzed the spatial distribution of the rest two subtypes of astrocytes: Astro1 (S1pr1) and Astro2 (Tspan7). We observed that, compared to the control mice, the Astro2 subpopulation in the HSD mice exhibited a gradual decrease in density toward the layer of pyramidal cells, whereas the Astro1 subpopulation showed no significant differences between the two groups (Figure 1M,N). Furthermore, the elevation of phosphorylation of the transcription factors NF‐κB and STAT3, closely associated with astrocyte‐mediated pro‐inflammatory processes, was also detected in the hippocampal astrocytes of HSD mice (Figure S2A,B, Supporting Information). Indeed, the expression of pro‐inflammatory cytokines, such as TNF‐α, IL‐6, IL‐1, IL‐2, IL‐17A, and IL‐3, but not the anti‐inflammatory cytokines are dramatically increased in HSD mice (Figure S2C–G, Supporting Information). As the reactivity of microglia but no change of oligodendrites was also detected in the hippocampus of HSD mice (Figure S3A,B, Supporting Information), we also analyzed the concentration of DAA‐like astrocyte toward the microglia. However, the Iba1+ microglia did not display a trend to migrate toward the center of the pyramidal cell layer, like reactive astrocytes or DAA‐like astrocytes (Figure S3C,D, Supporting Information). Additionally, there was no significant aggregation of DAA‐like astrocytes around reactive microglia (Figure S3E, Supporting Information). These data suggested that neurons, but not microglia may play an important role in mediating the DAA‐like astrocyte reactivity caused by HSD.

### 2.2 HSD‐Induced DAA‐Like Astrocytes Are Neuron‐Dependent

**Figure 2:**
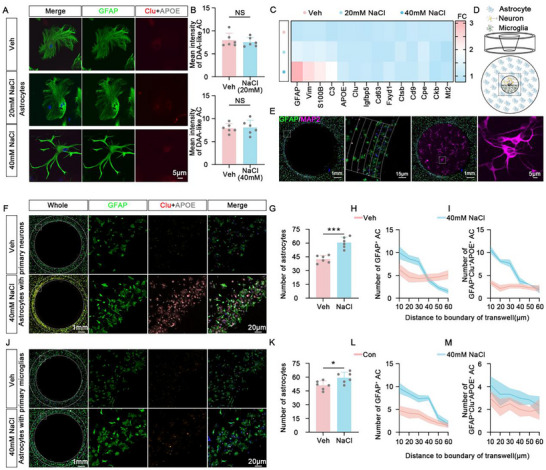
HSD‐induced DAA‐like astrocytes are neuron‐dependent. A) The representative immunofluorescence images for GFAP (green), Clusterin (red), and APOE (white) in primary astrocytes treated with 20 mM NaCl, and 40 mM NaCl. Blue staining represents DAPI. B) Mean intensity of Clu^+^APOE^+^ astrocytes in primary astrocytes treated with 20 mM NaCl (upper), and 40 mM NaCl (lower). *n* = 6 independent experiments. C) Transcriptome analysis to explore the differential expression of genes associated with reactive astrocytes and DAA astrocytes in primary astrocytes treated with Veh, 20 mM NaCl, and 40 mM NaCl. D) A diagram of the adapted transwell experiment for the co‐culture of primary neurons and astrocytes or primary microglia and astrocytes to analyze the aggregation of astrocytes surrounding the neurons and microglia. E) The representative immunofluorescence images for primary astrocytes (GFAP, green) and neurons (MAP2, purple) in the transwell experiment. Blue staining represents DAPI. F–I) The primary neurons, treated with vehicle or 40 mM NaCl were cultured in the central area of the transwell, while astrocytes were cultured in the surrounding cell culture plate outside the transwell walls. The representative immunofluorescence images for GFAP (green), Clusterin (red), and APOE (white) to visualize the DAA‐like astrocyte (F) surrounding the transwell walls. The number of astrocytes (G), the spatial distribution of GFAP^+^ astrocytes (H), and GFAP+Clu+APOE+ astrocytes (I) from 10 µm to 60 µm toward the boundary of the transwell were analyzed. J–M) The primary microglia, treated with vehicle or 40 mM NaCl were cultured in the central area of the transwell, while astrocytes were cultured in the surrounding cell culture plate outside the transwell walls. The representative immunofluorescence images for GFAP (green), Clusterin (red), and APOE (white) to visualize the DAA‐like astrocyte (J) surrounding the transwell walls. The number of astrocytes (K), the spatial distribution of GFAP+ astrocytes (L), and GFAP+Clu+APOE+ astrocytes (M) from 10 to 60 µm toward the boundary of the transwell were analyzed. *n* = 6 independent experiments.

To further confirm whether the neuron is indeed implicated in the HSD‐induced DAA‐like astrocyte reactivity, we treated the primary cultured neurons with 20 and 40 mm NaCl. Then, we co‐cultured these neurons with naïve primary astrocytes. Consistent with our in vivo data, astrocytes co‐cultured with HSD (20 mm)‐treated neurons for either 3 days or 7 days exhibited a significantly reactive state (Figure S4A–C, Supporting Information). However, direct HSD (20 mm, even 40 mm) treatment did not induce the formation of DAA‐like astrocytes (**Figure** **2**A,B). At the transcriptome level, treatment of primary astrocytes with 40 mm NaCl upregulated spectrum‐reactive astrocyte genes (GFAP, Vim) and classical neuroinflammation‐relative reactive astrocyte genes (S100B, C3). However, apart from the previously mentioned GFAP and Vim, which are reactive features associated with DAA astrocyte‐related genes, other DAA astrocyte‐related genes (Clu, Igfbp, Cd63, Fxyd1, Ctsb, Cd9, Cpe, Ckb, MT2) showed no significant changes (Figure 2C). To further elucidate the role of neurons in HSD‐induced DAA‐like astrocyte, we employed an adapted transwell apparatus. In this setup, the transwell bottom is attached to the cell culture plate, allowing for the culture of neurons or microglia in the central area of the transwell, while astrocytes are cultured in the surrounding cell culture plate outside the transwell walls (Figure 2D,E). We observed that the transwell containing high‐salt‐treated neurons was surrounded by a significant accumulation of GFAP‐positive astrocytes, including many GFAP^+^Clu^+^APOE^+^ DAA‐like astrocytes (Figure 2F–I). In contrast, while high salt‐treated microglia indeed induced aggregation of GFAP‐positive astrocytes, the transwell containing the high salt‐treated microglia was not surrounded by DAA‐like astrocytes (Figure 2J–M). These findings further substantiate that neurons, rather than microglia, play a pivotal role in the high‐salt‐induced formation of DAA‐like astrocytes.

### 2.3 Neuronal Tau Pathology Is Essential for HSD‐Induced Reactivity of DAA‐Like Astrocytes

**Figure 3:**
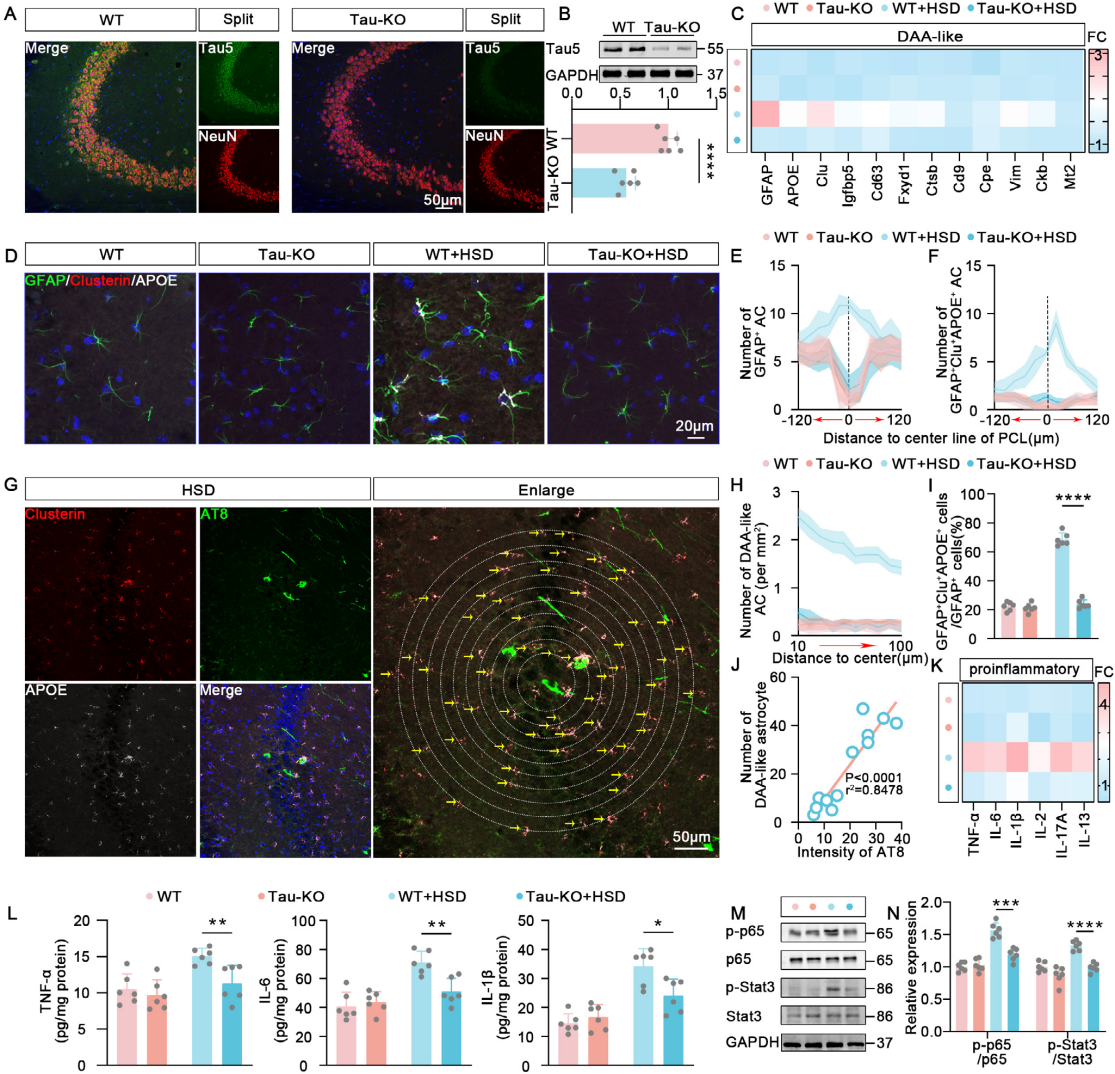
Neuronal Tau pathology is essential for HSD‐induced reactivity of DAA‐like astrocytes. A) The representative immunofluorescence images for Tau5 (green) and NeuN (red) in the hippocampal CA3 region of wild‐type (WT) and Tau‐KO mice. Blue staining represents DAPI. B) Immunoblot of Tau5 and GAPDH in the hippocampus of WT mice and Tau‐KO mice. *n* = 6 mice for each group. C) qPCR to examine the characterized genes of DAA‐like astrocytes in astrocytes sorted by magnetic beads from the hippocampus of WT mice, Tau‐KO mice, WT + HSD mice, and Tau‐KO + HSD mice. *n* = 6 mice for each group. D) Immunofluorescence for GFAP (green), Clusterin (red), and APOE (white) to label DAA‐like astrocytes in the hippocampus of WT mice, Tau‐KO mice, WT + HSD mice, and Tau‐KO + HSD mice. Blue staining represents DAPI. E,F) The spatial distribution of GFAP^+^ astrocytes (E) and GFAP^+^Clu^+^APOE^+^ astrocytes (F) toward the center of the pyramidal cell layer. *n* = 6 hippocampal slices for each group. G) The representative immunofluorescence images for AT8 (green), Clusterin (red), and APOE (white) in the hippocampal CA3 region of HSD‐treated WT mice. Blue staining represents DAPI. H) The spatial distribution of GFAP^+^Clu^+^APOE^+^ astrocytes from 10 to 100 µm to the edge of the tau phosphorylation region. I) The proportion of GFAP^+^Clu^+^APOE^+^ astrocytes among GFAP^+^ astrocytes in different groups. J) Correlation analysis between AT8 fluorescence intensity and the number of GFAP+Clu+APOE+ astrocytes. K) qPCR to examine the mRNA levels of TNF‐α, IL‐6, IL‐1β, IL‐2, IL‐17A, and IL‐13 in astrocytes sorted by magnetic beads from the hippocampus of WT mice, Tau‐KO mice, WT + HSD mice, and Tau‐KO + HSD mice. *n* = 6 mice for each group. L) ELISA analysis to examine the levels of TNF‐α, IL‐6, and IL‐1β in astrocytes sorted by magnetic beads from the hippocampus of WT mice, Tau‐KO mice, WT + HSD mice, and Tau‐KO + HSD mice. *n* = 6 mice for each group. M,N) Immunoblot (M) and the quantitative analysis (N) of the total and phosphorylation levels NF‐κB and STAT3 in the hippocampus of WT mice, Tau‐KO mice, WT + HSD mice, and Tau‐KO + HSD mice. *n* = 6 mice for each group.

Given the significant tau pathology observed in the CA3 region of the hippocampus in HSD mice,^[^ ^8^ ^]^ relative to CA1 and DG (Figures S5C and S7A,C, Supporting Information), and our previous findings demonstrating that tau pathology in CA3 neurons can induce reactivity of surrounding astrocytes, we hypothesize that neuronal tau pathology is the primary driver of DAA‐like astrocyte aggregation around CA3 pyramidal neurons in HSD mice. Indeed, tau pathology is prominent in CA3 neurons (Figure S5A–D, Supporting Information), where DAA‐like astrocytes are prone to accumulate. To confirm the critical role of tau pathology, we treated the Tau‐KO mice (**Figure** **3**A,B) with HSD. After sorting astrocytes by magnetic beads, we performed qPCR analysis for DAA genes in these mice. The expression of DAA genes was dramatically reduced in HSD‐treated Tau‐KO mice compared to the HSD treated wild type mice (Figure 3C). Immunofluorescence data further confirmed the decrement of reactive astrocytes and DAA‐like astrocytes in HSD Tau‐KO mice (Figure 3D–F). Importantly, to elucidate the spatial distribution between tau pathology and DAA‐like astrocytes, we analyzed the number of Clu^+^APOE^+^ astrocytes surrounding AT8^+^ neurons. We found that Clu^+^APOE^+^ astrocytes conspicuously aggregated around neurons with aberrant tau phosphorylation (Figure 3G–I). The fluorescence intensity of AT8 is significantly positively correlated with the numbers of surrounding DAA‐like astrocytes (Figure 3J), a correlation absent in the HSD‐treated Tau‐KO mice. Moreover, the expression of pro‐inflammatory cytokines, as well as the pro‐inflammatory transcription factors NF‐κB, and STAT3, was significantly downregulated in HSD‐treated Tau‐KO mice compared to HSD‐treated wild‐type mice (Figure 3K–M). We then investigated whether directly induced neuronal tau pathology could trigger the reactivity of DAA‐like astrocytes as seen in HSD mice. By injecting of tau mutant virus (AAV2/8‐hSyn‐MAPT(M)‐EGFP)(10^13^ TU mL^−1^, 200 nL) into the hippocampal CA3 region (Figure S6A, Supporting Information), we generated an artificially neuronal tau pathology mice model (Figure S6B, Supporting Information). We found that there was a significant aggregation of reactive astrocytes, particularly the aggregation of DAA‐like astrocytes surrounding the MAPT (M)‐infected area (Figure S6C–F, Supporting Information). By using laser capture microdissection, we isolated the regions with the aggregation of DAA‐like astrocytes (Figure S6G, Supporting Information) and found a significant upregulation of DAA‐associated genes in MAPT(M)‐infected regions (Figure S6H, Supporting Information). Simultaneously, levels of pro‐inflammatory cytokines were also upregulated in the regions infected with MAPT(M) (Figure S6I–L, Supporting Information). To further confirm the relationship between tau pathology and DAA astrocyte reactivity, we also examined the DAA astrocyte reactivity in the piriform cortex (Pir), a brain region previously reported to exhibit significant tau phosphorylation (Figure S7E, Supporting Information). We observed a similarly pronounced increase in DAA astrocyte reactivity in this region (Figure S7F, Supporting Information). Additionally, in two sodium‐sensitive hypothalamic regions, the subfornical organ (SFO) and the paraventricular nucleus (PVN), we also observed significant tau oligomer accumulation and enhanced DAA astrocyte reactivity (Figure S7G–J, Supporting Information). Collectively, these data strongly suggest that neuronal tau pathology is both essential and sufficient for HSD‐induced reactivity of DAA‐like astrocytes.

### 2.4 Activation of A1R Plays an Important Role in Mediating DAA‐Like Astrocyte Reactivity in HSD Mice

**Figure 4:**
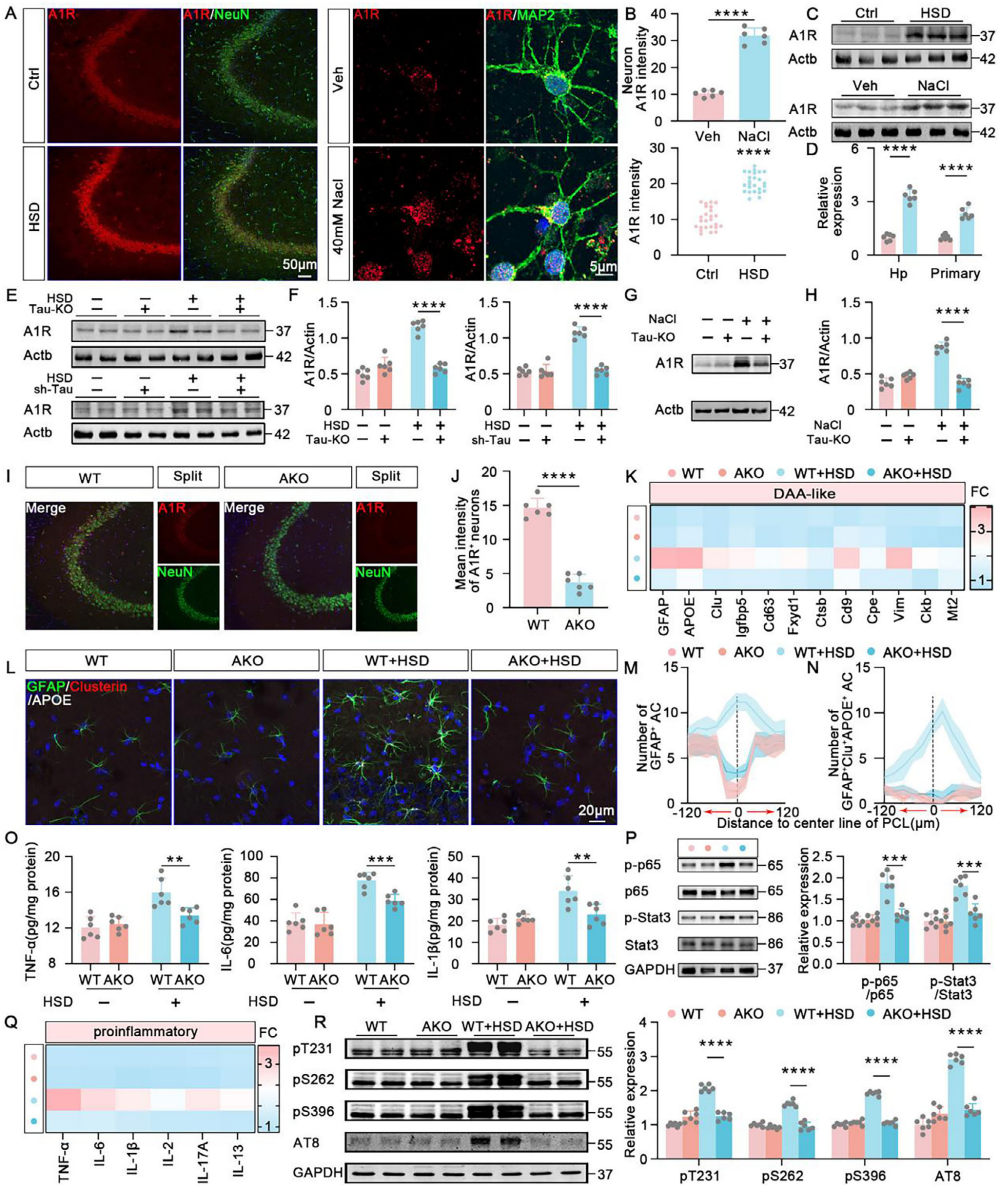
Activation of A1R plays an important role in Mediating DAA‐like Astrocyte Reactivity in HSD Mice. A) The representative immunofluorescence images for A1R (red) and NeuN (green) in the hippocampal CA3 region of Ctrl mice and HSD mice (left), and the primary cultured neurons treated with the vehicle or 40 mM NaCl (right). Blue staining represents DAPI. B) The quantification data of A1R intensity in panel A. *n* = 6 mice for each group. In vitro experiments, *n* = 6 independent experiments for each group. C) Immunoblot of A1R and Actb (loading control) in the hippocampus of Ctrl mice and HSD mice (upper), and the primary neurons treated with 40 mM NaCl or vehicles (lower). D) The quantification data for panel C. *n* = 6 mice for each group. E) Immunoblot of A1R and Actb in the hippocampus of WT mice, Tau‐KO mice, WT+HSD mice, and Tau‐KO + HSD mice, as well as scrambled shRNA (Ssh) mice, sh‐Tau mice, Ssh + HSD mice, and sh‐Tau + HSD mice. F) The quantification data for panel E. *n* = 6 mice for each group. G) Immunoblot of A1R and Actb in the primary neurons isolated from control fetal mice and Tau‐KO fetal mice, treated with 40 mM NaCl or vehicle, respectively. H) The quantification data for panel G. *n* = 6 independent experiments. I,J) The representative immunofluorescence images for A1R (green) and NeuN (red) (I) and the quantitative analysis for the A1R intensity (J) in the hippocampal CA3 region of A1R‐KO mice and WT mice. Blue staining represents DAPI. *n* = 6 hippocampal slices for each group. K) qPCR to examine the representative genes of DAA‐like astrocytes in astrocytes sorted by magnetic beads from the hippocampus of WT mice, A1R‐KO mice, WT + HSD mice, and A1R‐KO + HSD mice. *n* = 6 mice for each group. L) The representative immunofluorescence images for GFAP (green), Clusterin (red), and APOE (white) to label DAA‐like astrocytes in the hippocampus of WT mice, A1R‐KO mice, WT + HSD mice, and A1R‐KO + HSD mice. Blue staining represents DAPI. M,N) The spatial distribution of GFAP^+^ astrocytes and GFAP^+^Clu^+^APOE^+^ astrocytes toward the center of the pyramidal cell layer. *n* = 6 hippocampal slices for each group. O) ELISA analysis to examine the levels of TNF‐α (left), IL‐6 (middle), and IL‐1β (right) in astrocytes sorted by magnetic beads from the hippocampus of WT mice, A1R‐KO mice, WT + HSD mice, and A1R‐KO + HSD mice. *n* = 6 mice for each group. P) Immunoblot of total and phosphorylation levels NF‐κB, and STAT3 (left) and the quantitative analysis (right) in the hippocampus of WT mice, A1R‐KO mice, WT + HSD mice, and A1R‐KO + HSD mice. *n* = 6 mice for each group. Q) qPCR to examine the mRNA levels of TNF‐α, IL‐6, IL‐1β, IL‐2, IL‐17A, and IL‐13 in astrocytes sorted by magnetic beads from the hippocampus of WT mice, A1R‐KO mice, WT + HSD mice, and A1R‐KO + HSD mice. *n* = 6 mice for each group. R) Immunoblot of tau phosphorylation levels at different epitopes in the hippocampus of WT mice, A1R‐KO mice, WT + HSD mice, and A1R‐KO + HSD mice. *n* = 6 mice for each group.

In our previous research, we found that the high expression of neuronal A1R in AD plays a key role in tau pathology‐induced astrocyte reactivity.^[^ ^23^, ^32^ ^]^ Therefore, we first explored whether A1R also mediates DAA‐like astrocyte reactivity in HSD mice. We found that in high‐salt diet (HSD) mice and primary neurons treated with 40 mm NaCl, the expression levels of neuronal A1R were significantly upregulated (**Figure** **4**A–D). In contrast, in Tau‐KO mice treated with HSD, neuronal A1R expression was not elevated (Figure 4E,F). Consistently, bilateral hippocampal CA3 injection of Lenti‐U6‐shTau (10^13^ TU mL^−1^, 200 nL) virus in HSD mice, which alleviates tau pathology, also resulted in decreased neuronal A1R expression (Figure 4E,F). Additionally, in primary neurons from Tau‐KO mice treated with high salt, A1R expression was lower compared to that in wild‐type mice (Figure 4G,H). These results indicate that neuronal tau pathology mediates the abnormal upregulation of A1R induced by high salt. We then further investigated whether A1R signaling mediates DAA‐like astrocyte reactivity. We used A1R‐KO mice or bilateral hippocampal CA3 injection of Lenti‐hSyn1‐miR30‐shA1R‐P2A‐EGFP (10^13^ TU mL^−1^, 200 nL) to downregulate A1R expression (Figure 4I,J; Figure S9A,B, Supporting Information), followed by high‐salt diet treatment. In mice with downregulated A1R, the high‐salt diet did not induce the increased expression of DAA‐related genes (Figure 4K; Figure S9C, Supporting Information), nor did it provoke the aggregation of reactive and DAA‐like astrocytes around the pyramidal cell layer (Figure 4L–N; Figure S9D–F, Supporting Information). Moreover, in high‐salt diet, A1R‐KO, and sh‐A1R mice, the expression of inflammatory factors and pro‐inflammatory transcription factors NF‐κB and STAT3 were also downregulated (Figure 4O–Q; Figure S9G–I, Supporting Information). Interestingly, in a high‐salt diet, A1R‐KO and sh‐A1R mice, tau phosphorylation and tau oligomer levels were significantly reduced (Figure 4R; Figures S8A,C and S9J–L, Supporting Information). These results suggest that A1R signaling can not only act downstream of neuronal tau pathology to mediate HSD‐induced DAA‐like astrocyte reactivity but also elevate tau phosphorylation levels directly. These findings indicate that in HSD, there may be a vicious cycle between neuronal tau pathology and A1R upregulation.

### 2.5 A1R Activation Regulates Tau Pathology via the Cers1‐PP2A Signaling Pathway

**Figure 5:**
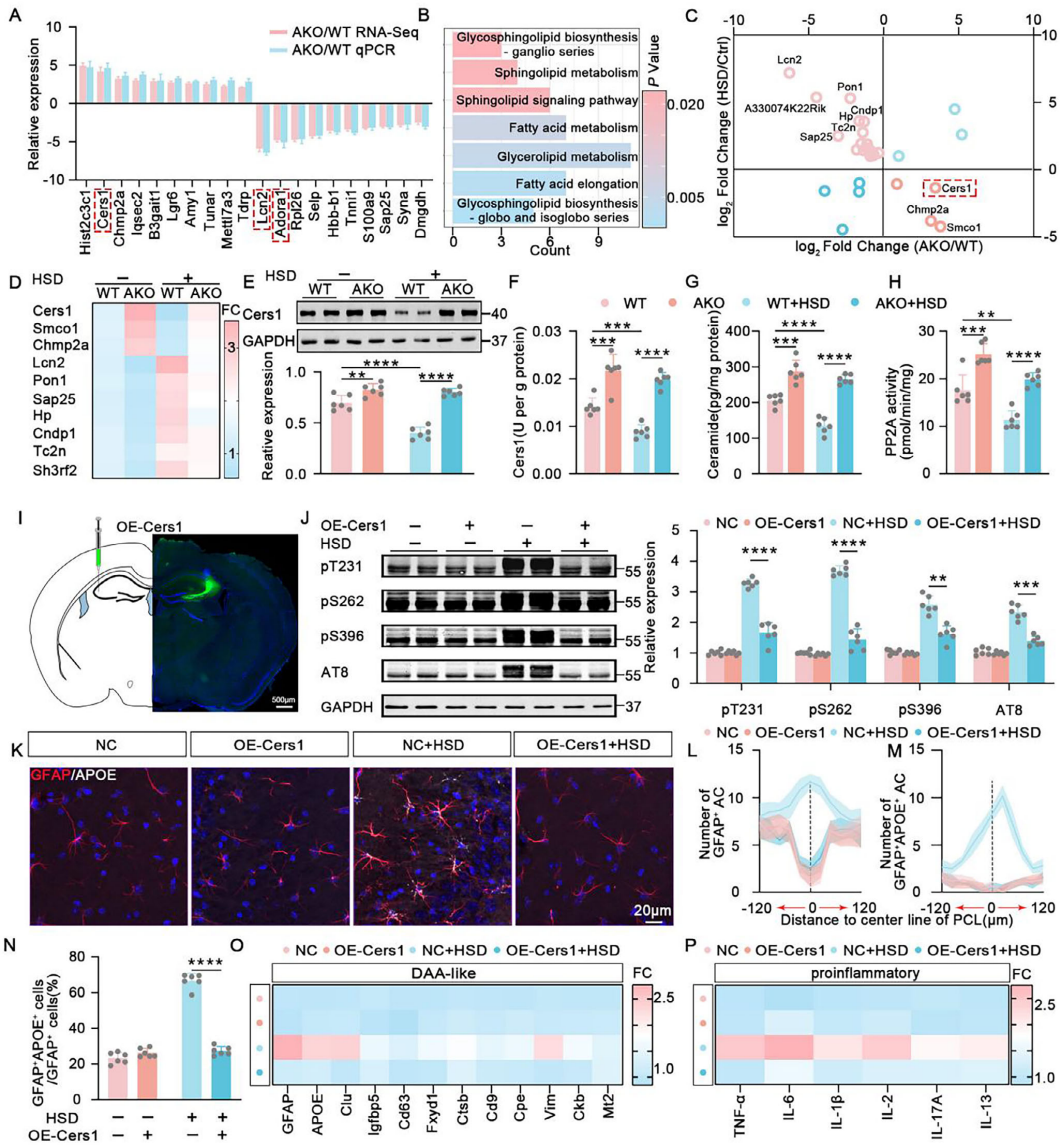
A1R Activation Regulates Tau pathology via the Cers1‐PP2A Signaling Pathway. A) Bar graph to show the top 10 upregulated and downregulated differentially expressed genes in the hippocampus of A1R‐KO mice compared to WT mice from the sequencing data (red bars) and qPCR validation (blue bars). B) GO enrichment analysis of deregulated genes in A1R‐KO mice compared with WT mice. C) Top 10 concordant deregulated genes in sequencing data from high‐salt diet (HSD) mice and AKO mice. D) qPCR to examine the top 10 concordant deregulated genes of panel C in the hippocampus of WT mice, A1R‐KO mice, WT + HSD mice, and A1R‐KO + HSD mice. *n* = 6 mice for each group. E) Immunoblot of Cers1 and GAPDH (upper) and the quantification data (lower) in the hippocampus of WT mice, A1R‐KO mice, WT + HSD mice, and A1R‐KO + HSD mice. *n* = 6 mice for each group. F,G) ELISA analysis to examine the levels of Cers1 (F) and ceramide (G) in the hippocampus of WT mice, A1R‐KO mice, WT + HSD mice, and A1R‐KO + HSD mice. *n* = 6 mice for each group. H) The enzyme activity of PP2A in the hippocampus of WT mice, A1R‐KO mice, WT + HSD mice, and A1R‐KO + HSD mice. *n* = 6 mice for each group. I) Diagram (left) and representative confocal image (right) to visualize the AAV2/8‐hSyn‐Cers1‐EGFP infection for Cers1 overexpression (OE‐Cers1). Blue staining represents DAPI. J) Immunblot of tau phosphorylation levels at different epitopes in the hippocampus of OE‐NC (NC) mice, OE‐Cers1 mice, NC + HSD mice, and OE‐Cers1 + HSD mice. *n* = 6 mice for each group. K) The representative immunofluorescence images for GFAP (red) and APOE (white) to label DAA‐like astrocytes in the hippocampus of NC mice, OE‐Cers1 mice, NC + HSD mice, and OE‐Cers1 + HSD mice. Blue staining represents DAPI. L,M) the spatial distribution of GFAP^+^ astrocytes (L) and GFAP^+^APOE^+^ astrocytes (M) toward the center of the pyramidal cell layer. *n* = 6 hippocampal slices for each group. N) The proportion of GFAP^+^APOE^+^ astrocytes among GFAP^+^ astrocytes. O) qPCR to examine the representative markers of DAA‐like astrocytes in astrocytes sorted by magnetic beads from the hippocampus of NC mice, OE‐Cers1 mice, NC + HSD mice, and OE‐Cers1 + HSD mice. *n* = 6 mice for each group. P) qPCR to examine the levels of TNF‐α, IL‐6, IL‐1β, IL‐2, IL‐17A, and IL‐13 in astrocytes sorted by magnetic beads from the hippocampus of NC mice, OE‐Cers1 mice, NC + HSD mice and OE‐Cers1 + HSD mice. *n* = 6 mice for each group.

Next, we investigated how A1R signaling regulates tau phosphorylation levels. By analyzing hippocampal sequencing data from AKO mice and control mice, we identified a set of 20 significantly altered genes, which we validated via qPCR (**Figure** **5**A). Gene set enrichment analysis of these differentially expressed genes revealed their close association with sphingolipid metabolism, sphingolipid signaling pathways, and glycosphingolipid biosynthesis (Figure 5B). Further comparative analysis of sequencing data from high‐salt diet (HSD) mice versus control mice and AKO mice versus control mice revealed several significantly differentially expressed genes that were inversely regulated in these two datasets, including Cers1, Chmp2a, Smco1, Lcn2, Pon1, Sap25, Hp, Cndp1, Tc2n, and Sh3rf2 (Figure 5C). We examined the expression of these genes in HSD‐treated wild‐type mice, A1R‐KO mice, and control mice, finding that Cers1 was significantly reduced in HSD‐treated wild‐type mice but markedly elevated following A1R‐KO (Figure 5D). Cers1 encodes a ceramide synthase, which catalyzes the synthesis of ceramide.^[^ ^33^ ^]^ Ceramide has been widely reported to activate PP2A and induce dephosphorylation.^[^ ^34^, ^35^ ^]^ We hypothesized that Cers1 might be a key downstream molecule of A1R in regulating tau pathology. Further examination of Cers1 and ceramide expression levels revealed that both were significantly upregulated in AKO mice, downregulated in HSD wild‐type mice, and partially restored in HSD‐treated AKO mice (Figure 5E–G). Additionally, PP2A enzymatic activity assays showed that PP2A activity was significantly reduced in the hippocampus of HSD‐treated wild‐type mice but restored to normal levels in HSD‐treated AKO mice (Figure 5H). We indeed detected the activation of Cdk5 in our HSD model, which is consistent with previous studies. We found that in A1R‐KO mice subjected to a high‐salt diet, neither the activity of cdk5 nor the levels of cdk5r1 (p35) were restored (Figure S9M, Supporting Information). Based on these findings, we propose that abnormal Cers1‐PP2A signaling may play a crucial role in A1R‐mediated tau pathology induced by HSD. Overexpression of Cers1 in the bilateral hippocampal CA3 region of mice via AAV2/8‐hSyn‐Cers1‐EGFP (10^13^ TU mL^−1^, 200 nL) injection (Figure 5I), followed by HSD treatment, significantly reduced tau phosphorylation and tau oligomer levels (Figure 5J; Figure S8B,D). Moreover, characteristic genes of DAA‐like astrocytes were significantly downregulated after Cers1 overexpression (Figure 5O), accompanied by a reduced aggregation of reactive and DAA‐like astrocytes toward the pyramidal cell layer (Figure 5K–N). Consistently, pro‐inflammatory cytokine levels were significantly lower in HSD‐treated oe‐Cers1 mice compared to HSD wild‐type mice (Figure 5P). In summary, these results indicate that in HSD mice, abnormal upregulation of A1R signaling can inhibit the ceramide‐PP2A axis, leading to abnormal tau phosphorylation.

### 2.6 Neuron‐Derived PAPD (Proteins Associated with Promoting DAA‐Like Astrocyte Reactivity) to Induce DAA‐Like Astrocyte Reactivity

**Figure 6:**
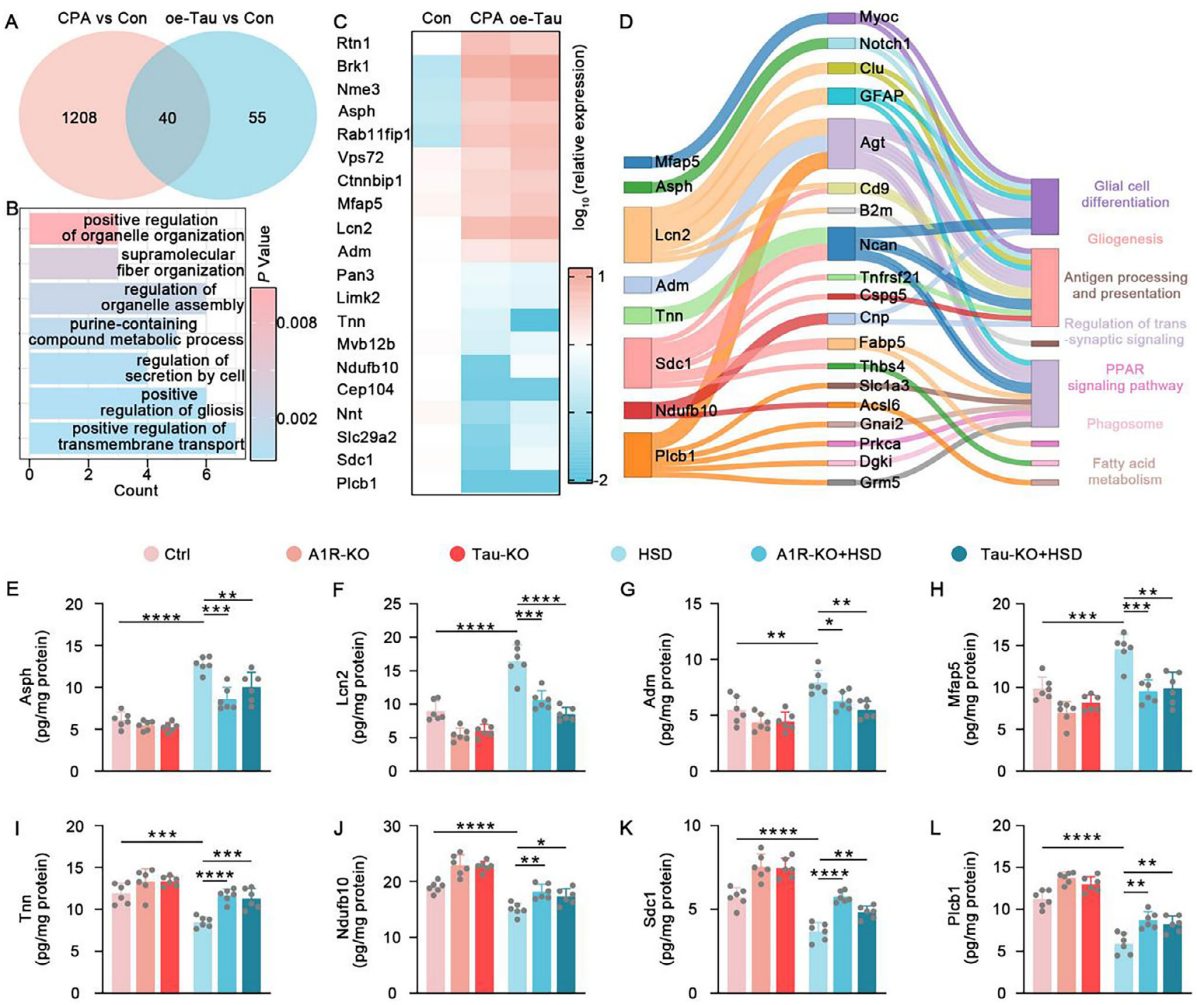
Neuron‐derived PAPD (Proteins Associated with Promoting DAA‐like Astrocyte Reactivity) to Induce DAA‐like Astrocyte Reactivity. A) Venn diagram showing the intersection of the secretome datasets from the CPA group versus the control group and OE‐Tau group versus the control group. B) GO enrichment analysis for the 40 intersected proteins in panel A. C) Top 20 consistently deregulated proteins in both the CPA and oe‐Tau groups. D) Sankey diagram showing the relationships between the selected candidate molecules (left line) inducing DAA‐like astrocyte reactivity, the characteristic functions of DAA‐like astrocytes (right line), and the representative genes (middle line) corresponding to these characteristic functions. E–L) ELISA analysis to examine the levels of Asph (E), Lcn2 (F), Adm (G), Mfap5 (H), Tnn (I), Ndufb10 (J), Sdc1 (K), and Plcb1 (L) in the cerebrospinal fluid (CSF) collected from WT mice, A1R‐KO mice, Tau‐KO mice, WT + HSD mice, A1R‐KO + HSD mice and Tau‐KO + HSD mice. *n* = 6 mice for each group.

To further investigate how the vicious cycle formed by pathological Tau and abnormal upregulation of A1R signaling induces DAA‐like astrocyte reactivity, we treated the primary neurons with N6‐Cyclopentyladenosine (CPA)(5 mm, 12 h), a selective adenosine A1 receptor agonist, to induce abnormal upregulation of A1R signaling. We also infected primary neurons with pcSLenti‐EF1‐mCherry‐CMV‐MAPT (10^12^ TU mL^−1^, 72 h) lentivirus (oe‐Tau) to induce tau pathology. The establishment of the tau pathology model in primary neurons was verified by tau phosphorylation and tau oligomer formation (Figure S10A,B, Supporting Information). Subsequently, we collected the cell culture supernatants from the CPA‐treated group, oe‐Tau group, and control group for secretome analysis. We found 1248 differentially expressed proteins in the CPA group compared to the control group, 95 differentially expressed proteins in the oe‐Tau group compared to the control group, and 40 common differentially expressed proteins between both groups (**Figure** **6**A). Enrichment analysis suggested that the functions of these 40 common proteins mainly involves in the organelle organization, cell secretion, gliosis, and transmembrane transport (Figure 6B). Next, we selected 20 proteins, which were commonly deregulated in both the CPA and oe‐Tau groups, as candidate molecules for inducing DAA‐like astrocyte reactivity (Figure 6C). Transcriptomic analysis of DAA‐like astrocytes showed significant associations with pathways including gliogenesis, glial cell differentiation, PPAR signaling, antigen processing and presentation, fatty acid metabolism, phagosome formation, and regulation of trans‐synaptic signaling (Figure 6D). Through interaction analysis of these 20 proteins with DAA‐related genes, we identified eight secreted proteins closely related to DAA‐like astrocyte characterization: Asph, Lcn2, Adm, Mfap5, Tnn, Ndufb10, Sdc1, and Plcb1. We termed these proteins as PAPD (proteins associated with promoting DAA‐like astrocyte reactivity) (Figure 6D).

We then collected cerebrospinal fluid (CSF) from control mice, A1R‐KO mice, Tau‐KO mice, and their respective high‐salt diet (HSD) models to examine the PAPD with ELISA. The results showed that Asph, Lcn2, Adm, and Mfap5 levels were significantly increased in the HSD mice but significantly decreased after A1R or Tau knockout. Conversely, Tnn, Ndufb10, Sdc1, and Plcb1 levels were significantly decreased in the HSD mice but improved after A1R or Tau knockout (Figure 6E–L). In summary, these results suggest that in the HSD model, neuronal tau pathology and A1R upregulation modulate the release of PAPD which in turn induces DAA‐like astrocyte reactivity.

### 2.7 Neuron‐Derived Lcn2 Is a Key Factor in Inducing DAA‐Like Astrocyte Reactivity

**Figure 7:**
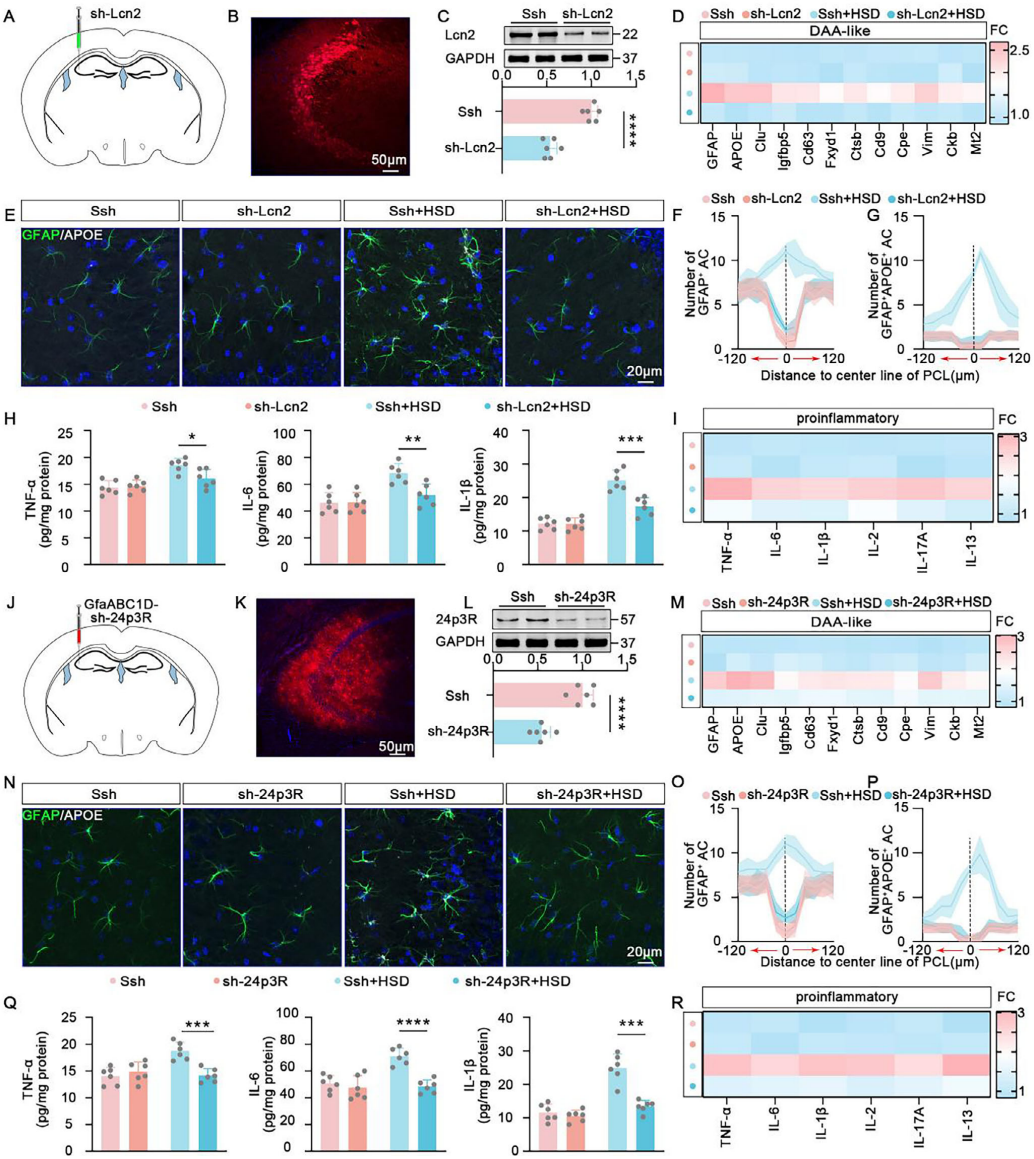
Neuron‐derived Lcn2 is a Key Factor in Inducing DAA‐like Astrocyte Reactivity. A,B) Diagram (A) and representative confocal image (B) visualize the virus rAAV2/9‐hSyn1‐miR30‐shLcn2‐P2A‐mCherry (sh‐Lcn2) injection site. C) Immunblot to assess the efficacy of sh‐Lcn2 lentivirus infection. Top: representative blot; Bottom: quantitative analysis. D) qPCR to examine representative markers of DAA‐like astrocytes in astrocytes sorted by magnetic beads from the hippocampus of Ssh mice, sh‐Lcn2 mice, Ssh + HSD mice, and sh‐Lcn2 + HSD mice. *n* = 6 mice for each group. E) The representative immunofluorescence images for GFAP (green) and APOE (white) to label DAA‐like astrocytes in the hippocampus of Ssh mice, sh‐Lcn2 mice, Ssh + HSD mice, and sh‐Lcn2 + HSD mice. Blue staining represents DAPI. F,G) The spatial distribution of GFAP^+^ astrocytes (F) and GFAP^+^APOE^+^ astrocytes (G) toward the center of the pyramidal cell layer. *n* = 6 hippocampal slices for each group. H) ELISA analysis to examine the levels of TNF‐α, IL‐6, and IL‐1β in astrocytes sorted by magnetic beads from the hippocampus of Ssh mice, sh‐Lcn2 mice, Ssh + HSD mice, and sh‐Lcn2 + HSD mice. *n* = 6 mice for each group. I) qPCR to examine the mRNA levels of TNF‐α, IL‐6, IL‐1β, IL‐2, IL‐17A, and IL‐3 of astrocytes sorted by magnetic beads in the hippocampus of Ssh mice, sh‐Lcn2 mice, Ssh + HSD mice, and sh‐Lcn2 + HSD mice. *n* = 6 mice for each group. J,K) Diagram (J) and representative confocal image (K) to visualize the AAV‐GfaABC1D‐sh‐24p3R‐mCherry (sh‐24p3R) infection site. L) Immunblot to assess the efficacy of sh‐24p3R virus infection. Top: representative blot; Bottom: quantitative analysis. M) qPCR to examine representative markers of DAA‐like astrocytes in astrocytes sorted by magnetic beads from the hippocampus of Ssh mice, sh‐24p3R mice, Ssh + HSD mice, and sh‐24p3R + HSD mice. *n* = 6 for each group. N) Immunofluorescence for GFAP (green) and APOE (white) to label DAA‐like astrocytes in the hippocampus of Ssh mice, sh‐24p3R mice, Ssh + HSD mice, and sh‐24p3R + HSD mice. Blue staining represents DAPI. O,P) The spatial distribution of GFAP^+^ astrocytes (O) and GFAP^+^APOE^+^ astrocytes (P) toward the center of the pyramidal cell layer. *n* = 6 hippocampal slices for each group. Q) ELISA analysis to examine the levels of TNF‐α, IL‐6, and IL‐1β in astrocytes sorted by magnetic beads from the hippocampus of Ssh mice, sh‐24p3R mice, Ssh + HSD mice, and sh‐24p3R + HSD mice. *n* = 6 for each group. R) qPCR to examine TNF‐α, IL‐6, IL‐1β, IL‐2, IL‐17A, and IL‐13 in astrocytes sorted by magnetic beads from the hippocampus of Ssh mice, sh‐24p3R mice, Ssh + HSD mice, and sh‐24p3R + HSD mice. *n* = 6 for each group.

Our previous studies have shown that neuronal tau pathology can promote the release of neuronal Lcn2 and induce reactive astrocyte reactivity. Among all the PAPD, Lcn2 exhibited the most significant changes. We observed that after one month of a high‐salt diet, neurons are the primary source of Lcn2 release, but after two months of the high‐salt diet, astrocytes also begin to release Lcn2 (Figure S11A,B, Supporting Information). These results suggest a temporal pattern in the source of Lcn2 release during the pathological progression of the high‐salt diet model. Early on, neurons are the main source, while astrocytes contribute at later stages. Therefore, in our one‐month high‐salt diet model, we chose to target neuron‐derived Lcn2 for intervention. We hypothesized that neuron‐derived Lcn2 plays a critical role in DAA‐like astrocyte reactivity in HSD mice. Therefore, we injected rAAV2/9‐hSyn1‐miR30‐shLcn2‐P2A‐mCherry (10^13^ TU mL^−1^, 200 nL) into the bilateral hippocampal CA3 regions of mice (**Figure** **7**A–C) and subjected them to HSD treatment. We found that knocking down neuronal Lcn2 resulted in a significant downregulation of DAA‐like astrocyte‐related genes in HSD mice (Figure 7D). Additionally, the trend of reactive and DAA‐like astrocytes migrating toward the pyramidal cell layer center was reduced (Figure 7E–G), accompanied by a corresponding decrease in pro‐inflammatory cytokine levels (Figure 7H,I).

**Figure 8:**
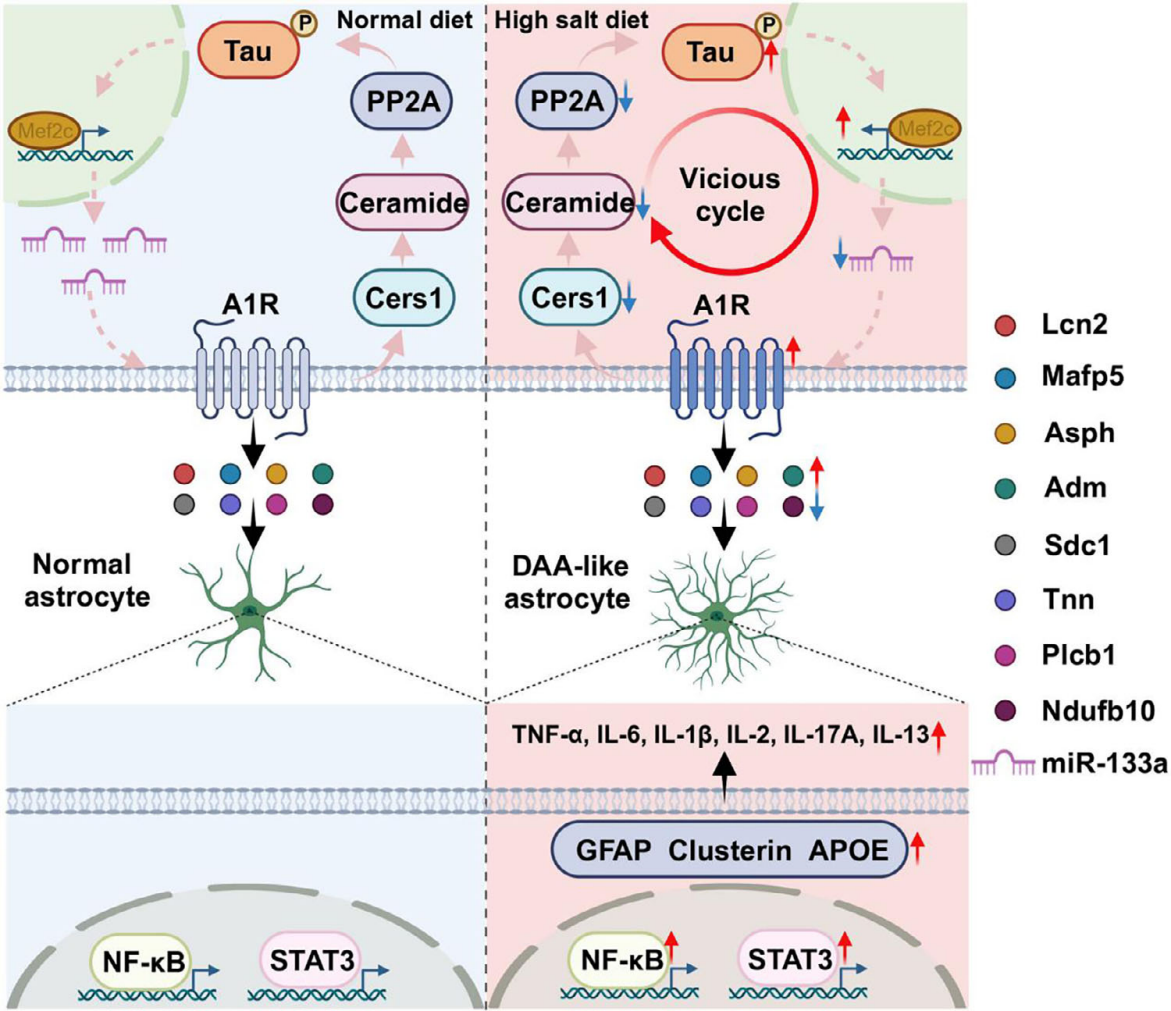
Schematic diagram of the current study. In the hippocampus of HSD mice, abnormally elevated levels of A1R lead to downregulation of Cers1, resulting in a decrease in ceramide levels, which inhibits the activity of PP2A and significantly increases tau phosphorylation. The dashed lines represent our previous research, where abnormally phosphorylated tau protein inhibits the transcription factor Mef2c, thereby reducing the transcription of miR‐133a‐3p, leading to increased A1R levels. Tau pathology and A1R signaling form a vicious cycle, inducing the abnormal release of neuronal PAPD (Lcn2, Mafp5, Asph, Adm, Sdc1, Tnn, Plcb1, and Ndufb10), ultimately triggering DAA‐like transcriptional changes in astrocytes (abnormal upregulation of GFAP, Clusterin, and APOE), activation of pro‐inflammatory transcription factors (NF‐κB and STAT3), and the release of pro‐inflammatory cytokines (TNF‐α, IL‐6, IL‐1β, IL‐2, IL‐17A and IL‐13).

Since Lcn2 primarily binds to the 24p3R receptor on astrocyte membranes, we specifically knocked down 24p3R in hippocampal astrocytes by injecting the virus AAV2/8‐GfaABC1D‐sh24p3r‐mCherry (Figure 7J–L). After one month, the HSD‐treated sh‐24p3r mice showed partial downregulation of DAA‐like astrocyte‐related genes compared to the HSD‐treated wild‐type mice (Figure 7M), along with a reduction in the migration trend of reactive and DAA‐like astrocytes toward the pyramidal cell layer center (Figure 7N‐P) and a decrease in pro‐inflammatory cytokine expression (Figure 7Q,R). We also injected recombinant Lcn2 protein (1 µg mL^−1^, 200 nL) into the bilateral hippocampal CA3 regions (Figure S12A,B, Supporting Information) and observed that it not only activated the characteristic genes of reactive astrocytes (GFAP and vimentin) but also partially activated the DAA‐like astrocyte characteristic genes (Clusterin, Ctsb, and APOE) (Figure S12C, Supporting Information). Immunofluorescence co‐staining showed significant aggregation of reactive astrocytes and DAA‐like astrocytes and migration toward the pyramidal cell layer center, similar to the HSD‐treated mice (Figure S12D–H, Supporting Information). Treatment of primary astrocytes with recombinant Lcn2 protein yielded consistent results, showing enhancement of astrocyte reactivity (Figure S12I,J, Supporting Information) and an increase in DAA‐like astrocyte characteristic genes (Clusterin, Ctsb, and APOE) (Figure S12K, Supporting Information). These findings indicate that Lcn2, as a key component of PAPD, plays an important role in HSD‐induced DAA‐like astrocyte reactivity (**Figure** **8**).

## 3 Discussion

One of the key observations was that HSD induced significant DAA‐like astrocyte reactivity, characterized by an increased number of GFAP^+^Clusterin^+^APOE^+^ astrocytes. Although previous studies have reported astrocyte reactivity in HSD mice,^[^ ^11^, ^12^, ^36^ ^]^ we are the first to discover the significant reactivity of DAA‐like astrocytes in HSD mice. Importantly, we found that these DAA‐like astrocytes exhibit significant aggregation around the CA3 pyramidal cell layer in the hippocampus of HSD mice. Extensive research indicates that astrocytes exhibit region‐specific reactivity and can form distinct reactive patterns depending on their environment and stimuli.^[^ ^37^, ^38^ ^]^ Previous studies suggest that DAA‐like astrocytes exhibit characteristics near plaques, it remains uncertain how these DAA‐like cells are attracted to the senile plaques. In our study, we demonstrated that the aggregation of DAA‐like astrocytes toward the pyramidal cell layer is primarily attributed to neuronal tau pathology. DAA‐like astrocytes are significantly reduced in tau‐knockout (Tau‐KO) mice, while these cells are markedly upregulated around neurons with aberrant tau phosphorylation. Previous studies have extensively reported the inductive role of tau pathology in astrocyte reactivity. Neuronal tau pathology mediates the interaction between neurons and astrocytes, resulting in the reactivity of astrocytes.^[^ ^23^ ^]^ In addition, astrocytic expression of 4R tau drives reactivity and dysfunction in astrocytes, resulting in phenotypes with diminished glutamate uptake capacity and increased sensitivity to oxidative stress.^[^ ^24^ ^]^ Higher astrocyte reactivity, as measured by plasma GFAP, was strongly linked with tau tangle aggregation and cortical thickness thinning in AD.^[^ ^39^ ^]^ Given the role of tau pathology in astrocyte reactivity, and in conjunction with our findings, we believe that tau pathology is sufficient to induce DAA‐like astrocyte reactivity independently.

Additionally, we found that A1R signaling plays a crucial mediating role in the reactivity of DAA‐like astrocytes. We noticed that downregulation of A1R significantly not only alleviated the formation of DAA‐like astrocytes but also reduced the tau pathology in vivo. As we previously reported the upregulation of A1R has been found to depend on tau pathology and is post‐transcriptionally regulated by Mef2c through miR‐133a‐3p.^[^ ^23^ ^]^ These data suggested a potentially vicious cycle between A1R upregulation and tau pathology, which in turn exacerbates tau phosphorylation and promotes DAA‐like astrocyte reactivity. Consistently, activation of A1R results in an ERK‐mediated increase in tau phosphorylation and its subsequent relocation to the cytoskeleton.^[^ ^40^ ^]^ Additionally, the tau inhibitor TRx 0237 has been demonstrated to substantially reverse the Aβ_25‐35_‐induced upregulation of A1R expression. Moreover, knocking down A1R can prevent the propagation of tau and phosphorylated tau.^[^ ^3^, ^4^ ^]^ tau pathology and A1R signaling jointly regulate glial reactivity, neuronal‐glial interactions, synaptic plasticity, and neuroinflammation.^[^ ^20^, ^21^, ^23^ ^]^ However, previous studies did not elucidate how A1R signaling regulates tau pathology in high‐salt mice. Our study reveals that the aberrant upregulation of A1R signaling in high‐salt diet mice leads to a significant downregulation of Cers1, potentially mediated by cAMP‐related transcriptional regulation. This, in turn, results in decreased levels of ceramides, which inhibits PP2A activity and exacerbates tau phosphorylation levels. Many studies have demonstrated the regulatory effect of ceramide on PP2A activity.^[^ ^34^, ^35^, ^41^ ^]^ Significant downregulation of Cers1 was observed in the hTau^MaptKO (Duke)^ mice that express the human MAPT gene. We found that tau phosphorylation levels were significantly reduced following Cers1 overexpression. These findings suggest the potential of ceramide as a therapeutic target for mitigating tau‐related neurodegeneration. Previous studies have demonstrated that a high‐salt diet exacerbates tau phosphorylation by activating cdk5. However, in our study, knocking down A1R did not alleviate cdk5 activation in HSD mice. Therefore, our findings suggest that the A1R‐Cers1‐PP2A signaling pathway mediates the exacerbation of tau phosphorylation induced by HSD independently of cdk5 activation. Given the significant upregulation of A1R signaling and notable tau phosphorylation have also been observed in Parkinson's disease (PD) and Amyotrophic lateral sclerosis (ALS),^[^ ^42^, ^43^, ^44^, ^45^ ^]^ we propose that this vicious cycle mechanism mediated by tau pathology and A1R signaling may extend beyond HSD and AD models, influencing the pathological mechanisms in various neurodegenerative disorders. Targeting both A1R signaling and tau pathology could therefore hold substantial promise for therapeutic strategies across a range of neurodegenerative diseases.

Moreover, the identification of PAPD, which includes Asph, Lcn2, Adm, Mfap5, Tnn, Ndufb10, Sdc1, and Plcb1, offers new insights into the molecular mechanisms underlying DAA‐like astrocyte reactivity. The differential expression of PAPD proteins in response to A1R signaling and tau pathology underscores their potential role in mediating neuroinflammatory responses. Specifically, the upregulation of Asph, Lcn2, Adm, and Mfap5 in the HSD mice and their subsequent downregulation upon A1R or Tau knockout suggest these proteins are integral to the pathophysiological changes induced by aberrant tau and A1R signaling. Among these, Lcn2 plays a particularly important role, as knockout of neuronal Lcn2 or knockout of the astrocyte 24p3R receptor significantly reduced the reactivity of DAA‐like astrocytes. Additionally, the injection of recombinant Re‐Lcn2 protein into the mouse hippocampus partially induced DAA‐like transcriptomic features. Lcn2 has been associated with neuroinflammation and glial reactivity in neurodegenerative diseases, our previous research found that neuronal A1R signaling promotes the release of Lcn2, leading to the reactivity of astrocytes.^[^ ^23^ ^]^ The Notch signaling pathway mediated by Asph and Mfap5 is considered to be closely associated with cell differentiation, fate determination, and proliferation.^[^ ^46^, ^47^ ^]^ In HSD mice, the upregulation of Mfap5 is also significant. Enhanced astrocyte reactivity and changes in the extracellular matrix (ECM) often co‐occur in AD. As an ECM‐associated protein, MFAP5 can influence ECM composition and structure, thereby affecting astrocyte morphology and activity. Additionally, MFAP5 is upregulated under conditions of tissue injury and inflammation, promoting an inflammatory environment that may shift astrocytes from a resting to a reactive state.^[^ ^48^, ^49^ ^]^ We believe that the upregulated PAPD proteins are responsible for inducing the reactivity of DAA‐like astrocytes. Conversely, the decreased levels of Tnn, Ndufb10, Sdc1, and Plcb1 in HSD mice, which improve following A1R or Tau knockout, suggest these proteins might have protective roles against DAA‐like astrocyte reactivity. The fibrinogen‐related domain of Tnn can bind and activate TLR4, which indicates that Tnn may play a role in inflammation and inflammatory diseases.^[^ ^50^ ^]^ A decrease in Ndufb10 levels reduces the assembly of mitochondrial respiratory chain complex I.^[^ ^51^ ^]^ In neurons the deficiency of respiratory chain complex I is a common mitochondrial disorder, often leading to the inhibition of oxidative phosphorylation and ATP deficiency, evoking neuroinflammation and neuronal death.^[^ ^52^ ^]^ Astrocytes largely rely on glycolysis for ATP production, besides,^[^ ^53^ ^]^ they possess an effective mitochondrial oxidation of fatty acids strengthening electron flux to complex III through electron transferring‐ubiquinone oxidoreductase bypassing complex I.^[^ ^54^ ^]^ However, in the astrocytes complex, I is responsible for the regulatory H_2_O_2_‐signaling to neurons sustaining synaptic transmission and cognitive performance.^[^ ^55^, ^56^ ^]^ Therefore the lack of NDufb10 protein can result in the disruption of astrocytic signaling pathways and cognition decline observed in HSD mice. Sdc1 functions as an integral membrane protein and participates in cell proliferation, cell migration, and cell‐matrix interactions via its receptor for extracellular matrix proteins.^[^ ^57^ ^]^ Plcb1 catalyzes the formation of inositol 1,4,5‐trisphosphate and diacylglycerol from phosphatidylinositol 4,5‐bisphosphate. This reaction uses calcium as a cofactor and plays an important role in the intracellular transduction of many extracellular signals.^[^ ^58^ ^]^ Our findings indicate that PAPD plays a critical role in the formation of DAA‐like astrocytes. Future research should explore the detailed mechanisms through which these proteins influence astrocyte reactivity and their potential as therapeutic targets in tau‐related neurodegenerative diseases.

Our study also has certain limitations. Although the degree of GFAP upregulation in reactive astrocytes typically parallels the severity of injury, this correlation is not always proportional, likely due to regional differences in astrocytes, including baseline GFAP levels. Additionally, studies have reported reduced GFAP immunoreactivity in astrocyte subpopulations within the cortex of mice following repeated injury and in the spinal cord of ALS mouse models, potentially due to caspase‐3‐mediated GFAP cleavage. Therefore, although GFAP is a widely used marker of astrocyte reactivity, it cannot independently capture the full spectrum of astrocytic responses across different pathological conditions. For example, using proliferation markers (Ki67, PCNA, and BrdU) and combining GFAP immunostaining with other widely present astrocyte markers (ALDH1L1, GS, ALDOC) can allow for a more accurate estimation of astrocyte numbers. Additionally, co‐labeling GFAP with other astrocyte reactivity markers (such as S100B and C3) can provide a more reliable assessment of their reactive state.

In conclusion, our study demonstrates that HSD can significantly induce DAA‐like astrocyte reactivity, with neuronal tau pathology and A1R signaling mediating this process. We also identify a series of PAPD, particularly Lcn2, whose release is significantly increased in the neurons burdened with tau pathology. Blocking neuronal Lcn2 release or knocking out the astrocyte 24p3R receptor can significantly alleviate DAA‐like astrocyte reactivity. Strategies targeting tau pathology, A1R signaling, and PAPD release may offer potential therapeutic targets for neuroinflammation and cognitive dysfunction induced by HSD.

## 4 Experimental Section

### Mice

The C57BL/6J mice (JAX: catalog no. 000664; RRID: IMSR_JAX: 000664) were obtained from the Jackson Laboratory (Bar Harbor, ME). The A1R knockout (AKO) mice (MGI ID: MGI: 4 940 034, RRID: IMSR_JAX:01 4161) were housed in the laboratory, generously provided by J. Schnermann at the National Institute of Diabetes and Digestive and Kidney Diseases, National Institutes of Health (NIDDK/NIH).^[^ ^23^, ^59^, ^60^ ^]^ Tau knockout (Tau KO) mice were also maintained in the laboratory, with genotyping conducted as previously described.^[^ ^61^ ^]^ All animals were kept on a 12‐h light/dark cycle in a temperature‐controlled room, with food and water available ad libitum. This study was approved by the Animal Care and Use Committee of Tongji Medical College (Wuhan, China).

### Cell Culture

Primary neurons were isolated from C57BL/6J mice and cultured as previously described.^[^ ^62^ ^]^ Cortices or hippocampi from embryos [embryonic day 16 (E16) and E17] were dissected, with the meninges removed. Tissues were digested in trypsin at 37 °C for 10 min and filtered through a 40‐µm cell strainer. The collected neurons were plated onto poly‐D‐lysine‐coated coverslips in six‐well plates containing plating medium (DMEM/F12 with 10% FBS and 1% penicillin/streptomycin) and incubated for 2–4 h. The media were then replaced with a maintenance medium (Neurobasal medium supplemented with 2% B‐27, 1 × GlutaMAX, and 1% penicillin/streptomycin), which was changed every 3 days with maintenance medium containing cytarabine (2 µm).

Primary astrocyte cultures were prepared from C57BL/6J mice as described previously.^[^ ^63^ ^]^ Briefly, mouse cortical tissues from postnatal day 1 (P1) were dissected after removing the meninges and incubated in 0.25% trypsin (Gibco) at 37 °C for 15 min. Dissociated cells were triturated with 0.25% fetal bovine serum (FBS), washed, and then centrifuged at 300 g for 10 min. The pellet was resuspended in DMEM/F12, passed through a 70µm nylon mesh, washed, and centrifuged again at 300 g for 10 min. Cells were plated on poly‐L‐lysine‐coated culture dishes at a density of 5–9 × 10^5^ cells mL^−1^ and allowed to adhere for one day in a 5% CO_2_ incubator at 37 °C, followed by dilution with glial medium (DMEM/F12 containing 10% FBS, 1% penicillin/streptomycin, and 0.2 mm L‐glutamine). Nonadherent cells were then removed, and a fresh glial medium was added. Adherent cells were maintained in the medium for 7 or 8 days, resulting in mixed glial cultures. To obtain higher‐purity astrocytes, primary cultured astrocytes were thoroughly agitated in an orbital incubator shaker at 350 rpm for 8 h. After agitation, the suspended cells in the culture medium, which included the purified microglia, were removed. The remaining attached cells were subcultured in DMEM/F12 medium with 10% FBS. Once the astrocytes reached confluence, they were passaged to generate pure astrocytic cultures. Cell purity was confirmed using monoclonal anti‐glial fibrillary acidic protein (GFAP), and only cultures with more than 95% GFAP‐positive cells were used for experiments.

An improved neuron‐astrocyte co‐culture model was developed. In this model, a 24 mm diameter, 0.4 µm pore‐sized Transwell (LABSELECT) in direct contact with a glass‐bottom cell culture dish was placed. Neurons were cultured inside the Transwell, while astrocytes were cultured on the surrounding glass bottom. Since astrocytes cannot penetrate the Transwell, this setup allowed to investigate the influence of astrocytes on the orientation and behavior of neurons.

### Virus and Recombinant Protein Injection

Mice were anesthetized, and a hole was generated in the skull above the hippocampal CA3 region. A total of 200 nL of rAAVs (10^13^ TU mL^−1^) and 200 nL of recombinant Lcn2 protein (1 µg mL^−1^) were stereotaxically injected at an infusion rate of 40 nL min^−1^ into the bilateral hippocampal CA3 (anterior/posterior, −2.0 mm; medial/lateral, ±2.5 mm; dorsal/ventral, −2.3 mm) using a Hamilton microsyringe. The needle was left in place for 10 min before being slowly withdrawn. Afterward, the wound was sutured, and the mice were allowed to recover.

### Magnetic Bead Sorting

A single‐cell suspension from neural tissue using a neural tissue dissociation kit was obtained, and the cell count was determined. The cell suspension at 300xg for 10 min was centrifuged. The supernatant was completely removed. The cell pellet in 80 µL buffer for every 10^7^ cells was resuspened. Ten liters of anti‐ACSA‐2 microbeads (Miltenyi Biotec) for every 10^7^ cells was added. Gently, it was mixed without vortexing and incubated at 2−8 °C for 15 min. The cells were washed by adding 1–2 mL buffer, centrifuged at 300xg for 10 min, and the supernatant was removed, resuspended in 500 µL buffer. The cell suspension was transferred to a separation column; cells bound to the magnetic beads will adhere to the column, while unbound cells will flow through. An appropriate amount of buffer was added, it was allowed to flow through completely, and the washing step was repeated two more times. Finally, a buffer was added to the separation column and quickly the plunger was pushed to elute the target cells.^[^ ^64^ ^]^

### Quantitative RT‐PCR

Total RNA was extracted using TRIzol reagent (Invitrogen, CA, United States) according to the manufacturer's instructions. One microgram of RNA was reverse‐transcribed for mRNA using a First‐Strand cDNA Synthesis Kit (TOYOBO, Osaka, Japan) or for miRNA using a miRcute Plus miRNA First‐Strand cDNA Kit (Tiangen, Beijing, China). Standard qPCR was performed on an ABI StepOnePlus real‐time quantitative PCR instrument using TB Green Premix Ex Taq II (Takara, Tokyo, Japan). The reaction conditions included pre‐denaturation at 95 °C for 3 min, followed by 40 cycles of denaturation at 95 °C for 5 s and annealing at 60 °C for 30 s. A melting curve analysis was subsequently performed, ranging from 60 to 95 °C, with temperature increments of 0.5 °C every 10 s. The primers used for RT‐PCR detection are listed in Table S2 (Supporting Information).

### Western Blot

Mice were euthanized, and the hippocampi were immediately isolated from the brains. The tissues were homogenized on ice using RIPA lysis buffer (Beyotime, Shanghai, China). Following a 10‐min boiling period, the samples were ultrasonically disrupted on ice 20 times. Protein concentrations were then measured with the BCA Protein Assay Reagent (Thermo Fisher Scientific, Illinois, USA). The proteins were separated on 10% SDS–polyacrylamide gel electrophoresis gels and transferred to nitrocellulose membranes (GE HealthCare Life Sciences, Loughborough, UK). After blocking with 5% nonfat milk for 60 min, the membranes were incubated with primary antibodies overnight at 4 °C, followed by washes with phosphate‐buffered saline (PBS)–Tween 20. Subsequently, the membranes were incubated with IRdye 800‐conjugated anti‐rabbit or anti‐mouse secondary antibodies (1:10000; Rockland Immunochemicals) for 1.5 h at room temperature. Protein bands were visualized using the Odyssey Imaging System (LI‐COR, Lincoln, NE, USA).^[^ ^65^ ^]^

### Immunocytochemistry

Immunocytochemistry staining was performed according to the manufacturer's instructions. After anesthetizing the mice with a mixture of ketamine (100 mg kg^−1^) and dexmedetomidine (0.5 mg kg^−1^), they were perfused with 1 × PBS, fixed with 4% (v/v) paraformaldehyde (PFA), and dehydrated with 30% sucrose for 24 h at 4 °C. Brain slices (30 µm thick) were obtained using a cryostat and rinsed three times with 1 × PBS. These slices were then treated with 3% hydrogen peroxide for 30 min to block endogenous peroxidase activity. After a 20‐min incubation with 0.1% Triton X‐100 to permeabilize the membranes, the slices were blocked with 5% bovine serum albumin (BSA) for 60 min. The brain slices were incubated with the primary antibody overnight at 4 °C. After washing with PBS, the slices were incubated with a biotinylated secondary antibody and a streptavidin‐labeled peroxidase solution for 1 h at room temperature. Subsequently, they were stained with 3,3′‐diaminobenzidine (DAB) reagent for 1–10 min at 37 °C. Following the staining, the brain slices were washed and dehydrated in a graded series of alcohol concentrations (75%, 80%, 95%, and 100%), rendered transparent in xylene, and mounted on glass slides. Digital images of all slices were captured using a Nikon Coolpix 5000 camera.

### Immunofluorescence Staining

The mice were anesthetized as described above and perfused with 0.9% (w/v) NaCl and 4% (v/v) PFA. Brain sections were cut with a cryostat to a thickness of 30 µm and then washed with PBS three times for 5 min each. Next, the membrane was permeabilized with 0.1% Triton X‐100 for 15 min. After blocking with 3% BSA for 60 min, the brain slices were incubated with the primary antibody at 4 °C overnight. After three washes with PBS, the brain slices were subsequently incubated with a fluorescent dye–conjugated secondary antibody for 1 h at room temperature in a dark environment. After washing, the nuclei were stained with 4′,6‐diamidino‐2‐phenylindole (DAPI) at room temperature for 10 min. Slices were imaged with a confocal laser scanning microscope LSM800 (Carl Zeiss, Oberkochen, Germany), and images were processed using ImageJ or Fiji software.

### Morris Water Maze

The Morris water maze test was conducted as previously described.^[^ ^59^ ^]^ A circular pool was filled with opaque water, and a hidden platform was placed just below the surface in one of the quadrants. The movement of the mice was recorded using a digital tracking system. In brief, the mice were trained to locate the hidden platform over seven consecutive days, with three trials each day. On the ninth day, a probe trial was conducted to assess spatial memory. The hidden platform was removed, and the mice were tested for their ability to find the previous platform location within 90 s, starting from the opposite quadrant. The escape latency to reach the former platform location, the swimming speed, and the percentage of time spent in each quadrant were measured and analyzed.

### Novel Location Test

The mice were first habituated to the testing environment, allowing them to freely explore and become familiar with the layout and features of the testing box. This habituation phase typically lasts for 10–15 min. Following habituation, two objects were placed in fixed positions within the testing environment, ensuring they were sufficiently spaced apart for the mice to distinguish between them. The mice were then allowed to freely explore the objects and their surroundings for 5–10 min, helping them establish a memory of the object locations. After the training session, the mice were removed from the testing environment and given a rest period, known as the delay period, which typically lasted from 1 to 24 h. Following the delay period, the mice were reintroduced to the testing environment, with one of the objects moved to a new location while the other object remained in its original position. The mice's exploration behavior toward the objects was recorded for a few minutes. The exploration times of the mice toward the moved object and the original object were calculated. Video recordings or automated tracking software were commonly used to analyze the mice's behavior. By comparing the exploration times of the moved object and the original object during the testing period, the mice's spatial memory abilities could be evaluated. Longer exploration times indicated that the mice were able to detect the change in object location and demonstrate new location memory.^[^ ^66^ ^]^

### T maze

Before each experiment, the T‐maze is thoroughly cleaned and disinfected to ensure a suitable and sanitary environment. In the days leading up to the experiment, the mice undergo a habituation phase where they are placed in the T‐maze and allowed to freely explore its different arms and paths. This phase typically lasts for 10–15 min and helps the mice become familiar with the maze's layout and features. Following habituation, the mice are trained to complete a specific task. They are placed in the starting area of the T‐maze, and a reward object (such as food or water) is placed in one of the arms. The mice must choose the correct path to reach the reward object within a specified time period. The training can be adjusted to increase difficulty by introducing more complex maze configurations or shorter time limits. After a certain number of training sessions, a testing phase is conducted. In this phase, the reward object is removed, and the mice must choose the correct path without the presence of a reward. The behavior of the mice during the testing phase, including path choices, errors, and completion time, is recorded. Video recordings or automated tracking software are often used to analyze the behavior of the mice. By comparing the performance of the mice during training and testing, their learning and memory abilities in the T‐maze task can be evaluated.^[^ ^67^ ^]^ Fewer errors and shorter completion times indicate that the mice have learned the correct path and possess spatial memory abilities.

### Long‐Term Potentiation (LTP) Recording

Mice were anesthetized, and coronal brain slices (300 µm thick) were prepared in artificial cerebrospinal fluid (ACSF) bubbled with 95% O_2_ and 5% CO_2_, containing 124 mm NaCl, 3.0 mm KCl, 2.0 mm CaCl_2_, 1.2 mm MgSO_4_, 1.25 mm KH_2_PO_4_, 26 mm NaHCO_3_, and 11 mm glucose, following previously described methods.^[^ ^59^, ^68^ ^]^ The slices were then immersed in oxygenated ACSF at 30 °C for at least 30 min before being transferred to a recording chamber filled with ACSF. Field excitatory postsynaptic potentials (fEPSPs) were recorded from CA3 neurons by stimulating mossy fibers. The MED64 system (Alpha Med Sciences, Tokyo, Japan) was used to record the fEPSPs. After a 30‐min stable baseline period, long‐term potentiation (LTP) was induced by tetanic stimulation consisting of three trains of 100‐Hz stimuli delivered at 30‐s intervals.

### Whole‐Cell Patch‐Clamp Recording

For whole‐cell patch‐clamp recordings,^[^ ^69^, ^70^, ^71^ ^]^ pipettes with a resistance of 5 MΩ were filled with an internal solution containing 140 mm potassium gluconate, 2 mm NaCl, 10 mm Hepes, 0.2 mm EGTA, 2 mm Mg‐ATP, and 0.3 mm Na‐GTP (290 mOsm). The voltage‐clamp solution included 17.5 mm CsCl, 0.05 mm EGTA, 10 mm Hepes, 2 mm MgATP, 0.2 mm GTP, and 5 mm QX‐314 (pH 7.4, 292 mOsm). To record mEPSCs, pyramidal neurons from the hippocampal CA3 region were held at −70 mV in the presence of 1 mm tetrodotoxin and 10 mm bicuculline. Electrophysiological data were filtered at 12 kHz and acquired at 10 kHz using ClampFit 10.2 software (Molecular Devices). MiniAnalysis software (Synaptosoft, Decatur, GA) was used to analyze mEPSC events with a threshold amplitude of 5 pA. Data analysis was performed using GraphPad Prism 8.0, and figures were refined using Adobe Illustrator CS6.

### Golgi Staining

Golgi staining was performed using an FD Rapid GolgiStain Kit (catalog no. PK401, FD Neuro Technologies Inc.) according to the manufacturer's instructions. Briefly, the mice were anesthetized and perfused with 0.9% NaCl for 1 min, after which the brains were quickly removed from the skull. Following rinses with double‐distilled water, the brains were immersed in impregnation solution (a mixture of equal amounts of solutions A and B) for 2 weeks at room temperature in the dark, with the solution being changed every 3 days. Subsequently, the brains were transferred to solution C and incubated at room temperature in the dark for 1 week. Brain slices were cut at a thickness of 100 µm using a vibrating blade microtome (Leica VT1000s) and then mounted on high‐adhesion glass slides with solution C. The brain sections were stained with a mixture of solutions D and E for 10 min. The slices were then washed, dehydrated with sequential ethanol solutions for 4 min each, and cleared in xylene three times for 4 min each. Images were captured using a Nikon Coolpix 5000 camera. Sholl analysis and dendritic spine analysis were performed using ImageJ or Fiji software, as previously described.^[^ ^23^, ^62^, ^72^ ^]^

### Sholl Analysis and Imaris

Sholl analysis was performed to assess the complexity of the astrocyte arbors and dendritic trees, following previously described methods.^[^ ^73^ ^]^ The dendritic spines were counted in two segments on the main branches, located 100 and 200 µm from the soma, as previously reported. 3D reconstructions were created using Imaris software.^[^ ^23^, ^74^ ^]^

### Laser Capture Microdissection

The hippocampal CA3 region was isolated from brain slices using laser capture microdissection according to previously described methods.^[^ ^23^, ^75^ ^]^ Mice were anesthetized as previously described, and each mouse's brain was dissected and prepared into 100 µm acute slices using a vibratome. The slices were stained with Hoechst live‐cell nuclear dye to determine tissue localization and were mounted onto 20 glass slides. Using the Leica LMD7 laser capture microdissection system, the hippocampal CA3 regions were selectively captured with a laser setting of 7.5 µm and a pulse set to 90 mW for 1 ms. RNA was then extracted from these selectively captured tissues and analyzed using qPCR.

### Extraction of Cerebrospinal Fluid

The cerebrospinal fluid (CSF) extraction procedure was referenced from previous studies.^[^ ^76^ ^]^ To begin, a sterile cotton swab is used to stretch the atlas and tighten the dura mater. A capillary glass siphon tube, not connected to a negative pressure device, is employed to puncture the dura mater. If penetration is challenging, gentle rotation of the glass tube can facilitate the process. Due to the proximity of the dura mater to the brainstem in mice, a noticeable breakthrough should be felt, accompanied by the entry of clear fluid into the glass tube. At this point, the position is locked, and movement of the capillary glass siphon tube is halted to avoid damage to the brainstem and its blood vessels, preventing potential bleeding and contamination of the CSF. Once the CSF ceases to rise in the tube, the needle is carefully withdrawn, and the outer wall of the glass tube is cleaned with a dry cotton swab before transferring the CSF to a 200 µL EP tube. Following the initial extraction, the capillary glass siphon tube is connected to a 1 mL sterile syringe to create a negative pressure device. A slight pull on the syringe plunger draws in a small amount of air, facilitating CSF transfer and plunger movement. The tip of the capillary glass siphon tube is reinserted into the medullary cistern through the puncture site, and the syringe plunger is slowly pulled to extract additional CSF. If the CSF no longer rises in the tube, adjustments to the glass tube's position may be made, or the extraction can be stopped, with the CSF being transferred to another 200 µL EP tube.

### Enzyme‐Linked Immunosorbent Assay

Ceramide and protein levels of Cers1, Asph, Lcn2, Adm, Mfap5, Tnn, Ndufb10, Sdc1, and Plcb1 were detected by homogenizing the hippocampus in buffer containing 50 mm NaCl, 10 mm tris‐HCl, 1 mm EDTA, 2% SDS, and 0.5 mm Na3VO4. The homogenates were boiled for 10 min and then centrifuged at 10 000g for 10 min. The final supernatants were used for ELISA, and the cell culture supernatant samples were treated according to the manufacturer's instructions.

### Statistical Analysis

All data are presented as means ± SEM and were analyzed using GraphPad Prism software (version 8). A two‐tailed Student's *t*‐test was used to assess variance between two groups, while differences among multiple groups were evaluated using one‐way or two‐way analysis of variance (ANOVA) followed by post hoc tests. Correlations were examined using a linear regression model and chi‐square test. Both *p* < 0.01 and *p* < 0.001 represent extremely significant differences.

## Conflict of Interest

The authors declare no conflict of interest.

## Author Contributions

T.‐Y.R., H.‐Z.H., and K.Z. contributed equally to this work. L.‐Q.Z. and D.L. designed and supervised the project, T.‐Y.R. and H.‐Z.H. performed the molecular biological experiments, and bioinformatic analysis, H.‐W.F. performed the electrophysiological recording, T.‐Y.R., H.‐Z.H., K.Z., and H.‐W.F. analyzed the data, T.‐Y.R., L‐Q.Z, and D. L. wrote the manuscript. T.‐Y.R. H.‐Z.H. J.Z., Z‐Y.G., H.Y.M., N.B., A.S., Y.‐M.L., L‐Q.Z, and D.L. revised and approved the manuscript.

## Supporting information



Supporting Information

## Data Availability

Research data are not shared.
