# Supplementary material for: Tau Pathology Drives Disease‐Associated Astrocyte Reactivity in Salt‐Induced Neurodegeneration
Source: Adv Sci (Weinh). 2025 Jan 24;12(11):2410799. doi: 10.1002/advs.202410799 (PMC11923866; doi:10.1002/advs.202410799)
Supplement: Supplementary file 1 — Supporting Information [file ADVS-12-2410799-s001.docx]

**Supplementary Materials for**

**Tau Pathology Drives Disease-Associated Astrocyte Activation in Salt-Induced Neurodegeneration**

Tong-Yu Rui *et al.*

Corresponding author: Ling-Qiang Zhu, zhulq@mail.hust.edu.cn; Dan Liu, liudan_echo@mail.hust.edu.cn

**The PDF file includes:**

Supplementary Figure S1 to S12

Supplementary Tables S1 to S3

Supplementary data full gels for all the Western blots in this study


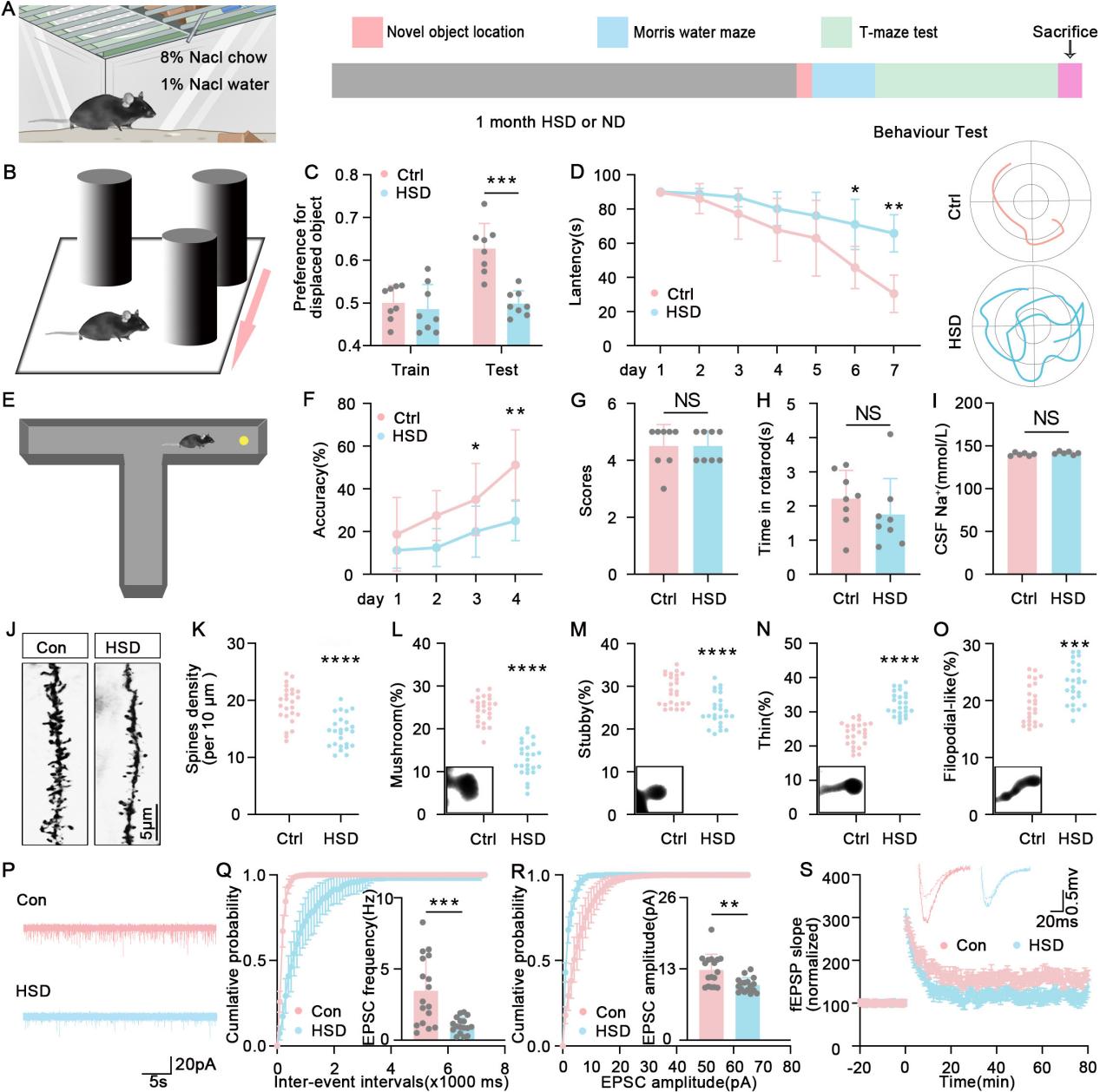


**Figure S1 High-salt diet impairs spatial memory, synaptic transmission and dendritic morphology.**

(A) Diagram of High-Salt Diet Model and Behavioral Tests. (B) Diagram of the Novel Location Test. (C) Preference for displaced object in Novel Location Test of Ctrl mice and HSD mice. n = 8 mice for each group. (D) Latency and the representative swimming traces on day 7 in Morris water maze of Ctrl mice and HSD mice. n = 8 mice for each group. (E) Diagram of the T-Maze test. (F) The accuracy of Ctrl mice and HSD mice obtaining food in the T-Maze test. n = 8 mice for each group. (G) Scores in Pole Test of Ctrl mice and HSD mice. n = 8 mice for each group. (H) Time on rotarod in Rotarod Test of Ctrl mice and HSD mice. n = 8 mice for each group. (I) The Na^+^ concentration of cerebrospinal fluid in Ctrl mice and HSD mice. n = 6 mice for each group. (J-O) The representative images of Golgi staining in the hippocampal CA3 region of Ctrl mice and HSD mice (J) and the quantitative analysis of spine density (K), percentages of mushroom-type spine (L), stubby-type spine (M), thin-type spine (N), filopodia-type spine (O). (P-R) Representative traces and quantitative analysis for the frequency and amplitude of mEPSCs from hippocampal CA3 region of Ctrl mice and HSD mice. n = 17 cells from 6 mice for each group. (S) The slope of field excitatory postsynaptic potential (fEPSP) of hippocampal CA3 region in Ctrl mice and HSD mice. n = 6 mice for each group.


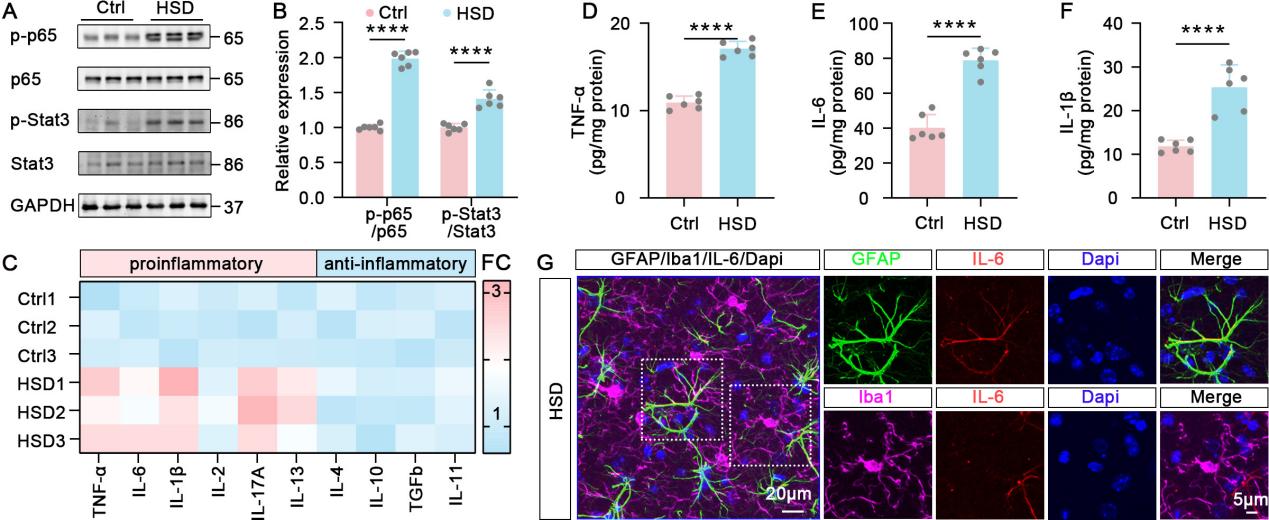


**Figure S2 The inflammatory responses in HSD mice.**

1. B) Immunoblot of the total and phosphorylation levels of NF-κB, STAT3 in the hippocampus of HSD mice and Ctrl mice. The representative blots were shown in A and the quantitative analysis was shown in B. n = 6 mice for each group. (C) qPCR to examine the levels of TNF-α, IL-6, IL-1β, IL-2, IL-17A, IL-13, IL-4, IL-10, TGFβ and IL-11 in astrocytes sorted by magnetic beads from the hippocampus of Ctrl mice and HSD mice. n = 6 mice for each group. (D-F) ELISA analysis to examine levels of TNF-α, IL-6 and IL-1β in astrocytes sorted by magnetic beads from the hippocampus of Ctrl mice and HSD mice. n = 6 mice for each group. (G) The representative immunofluorescence images for TNF-α (red) in astrocyte (GFAP, green) and microglia (Iba1, purple) in the hippocampal CA3 region of HSD mice. n = 6 hippocampal slices for each group.


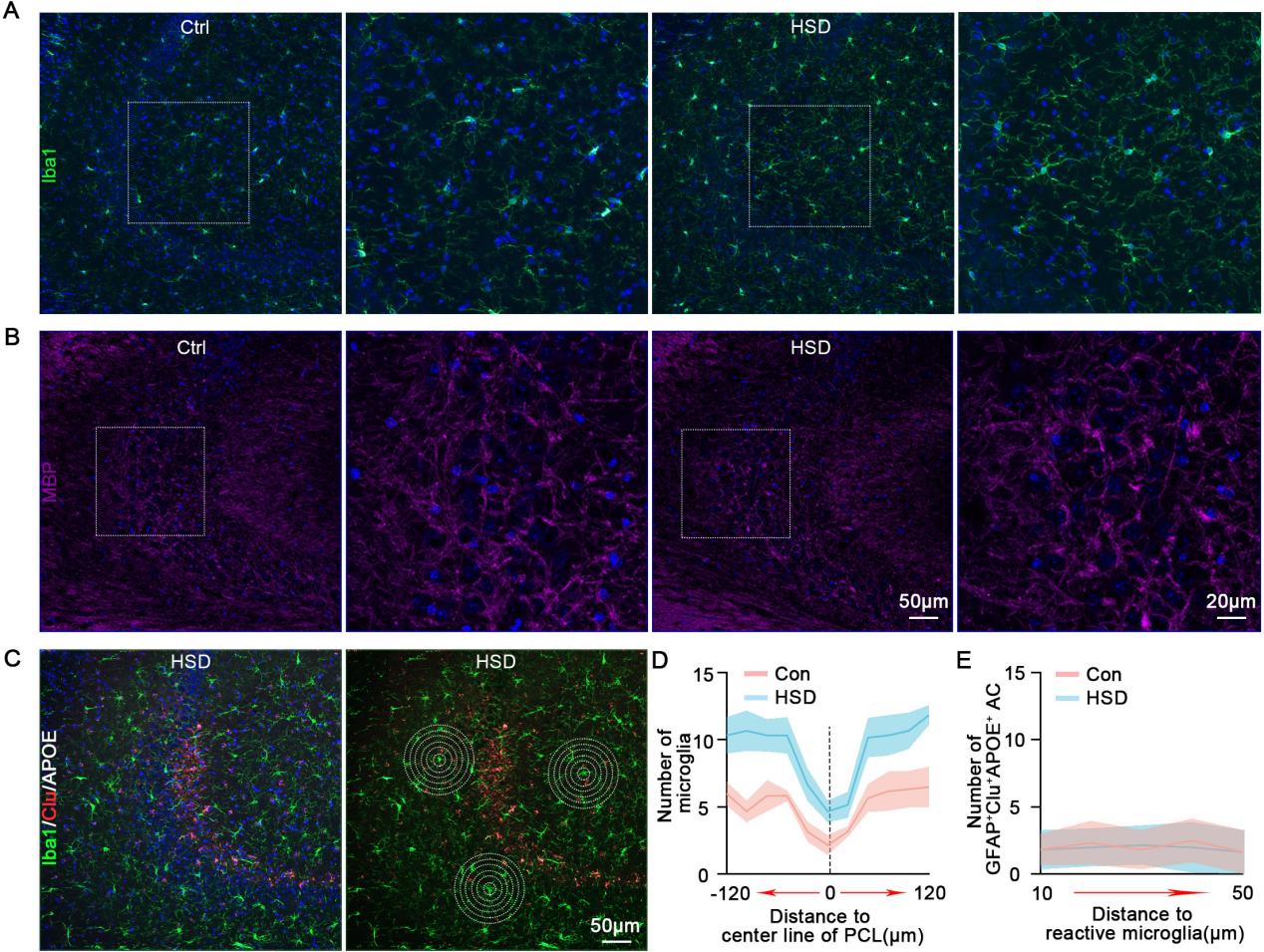


**Figure S3 HSD-induced DAA-like astrocytes are neither microglia- nor oligodendrocytes-dependent.**

1. B) The representative immunofluorescence images for the microglia (Iba1, green, panel A) and oliodendrocytes (MBP, purple, panel B) in the hippocampal CA3 region of Ctrl mice and HSD mice. Blue staining represents DAPI. (C) The representative immunofluorescence images for Iba1 (green), clusterin (red) and APOE (white) in the hippocampal CA3 region of HSD mice. Blue staining represents DAPI. A row of circles in the right panel indicate the distance to the Iba1^+^ microglia and a 10 μm interval is set between circles. (D) The spatial distribution of microglia toward the center of the pyramidal cell layer in the hippocampal CA3 region of Ctrl mice and HSD mice. n = 6 hippocampal slices for each group. (E) The spatial distribution of GFAP^+^Clu^+^APOE^+^ astrocytes toward the center of the reactive microglia. n = 6 hippocampal slices for each group.


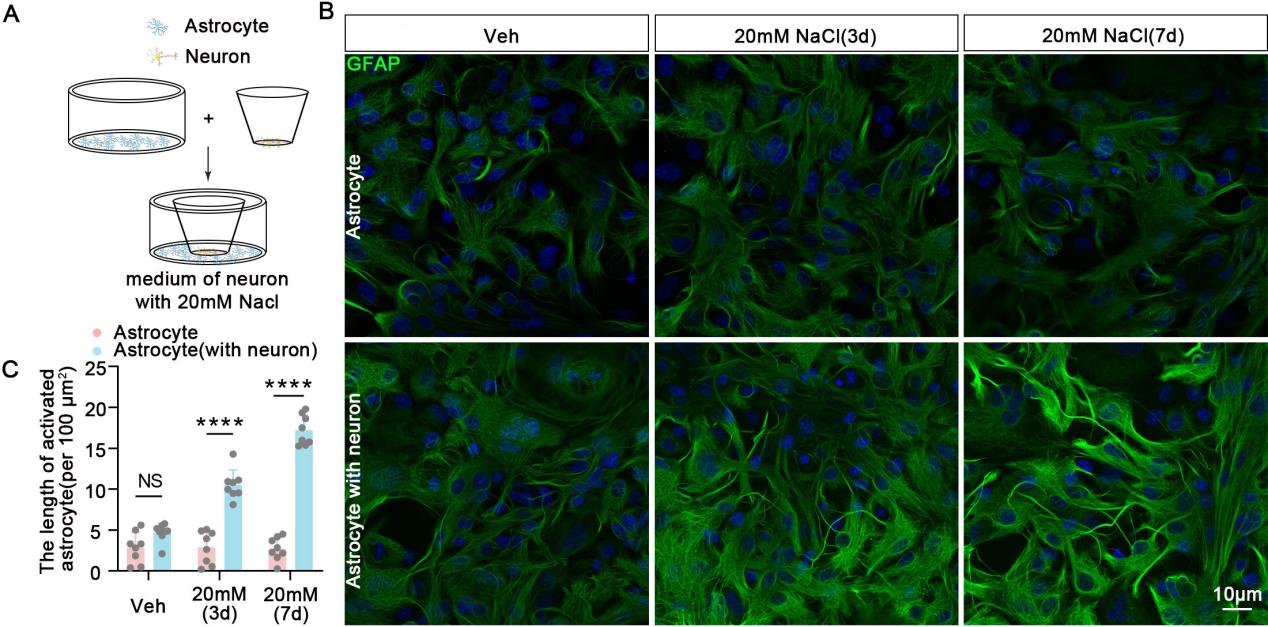


**Figure S4 High salt didn’t induce DAA-like astrocyte directly *in vitro*.**

1. Diagram for the co-culture of primary neurons and astrocytes and treated with 20 mM NaCl. (B) Immunofluorescence for GFAP (green) in astrocytes alone or co-cultured with neurons, under the vehicle and 20 mM NaCl treatments (3 days or 7 days), respectively. Blue staining represents DAPI. (C) The length of activated astrocyte in astrocytes cultured alone or co-cultured with neurons, under control conditions, 20 mM NaCl treatment (3 days), and 20 mM NaCl treatment (7 days), respectively. n = 6 independent experiments.


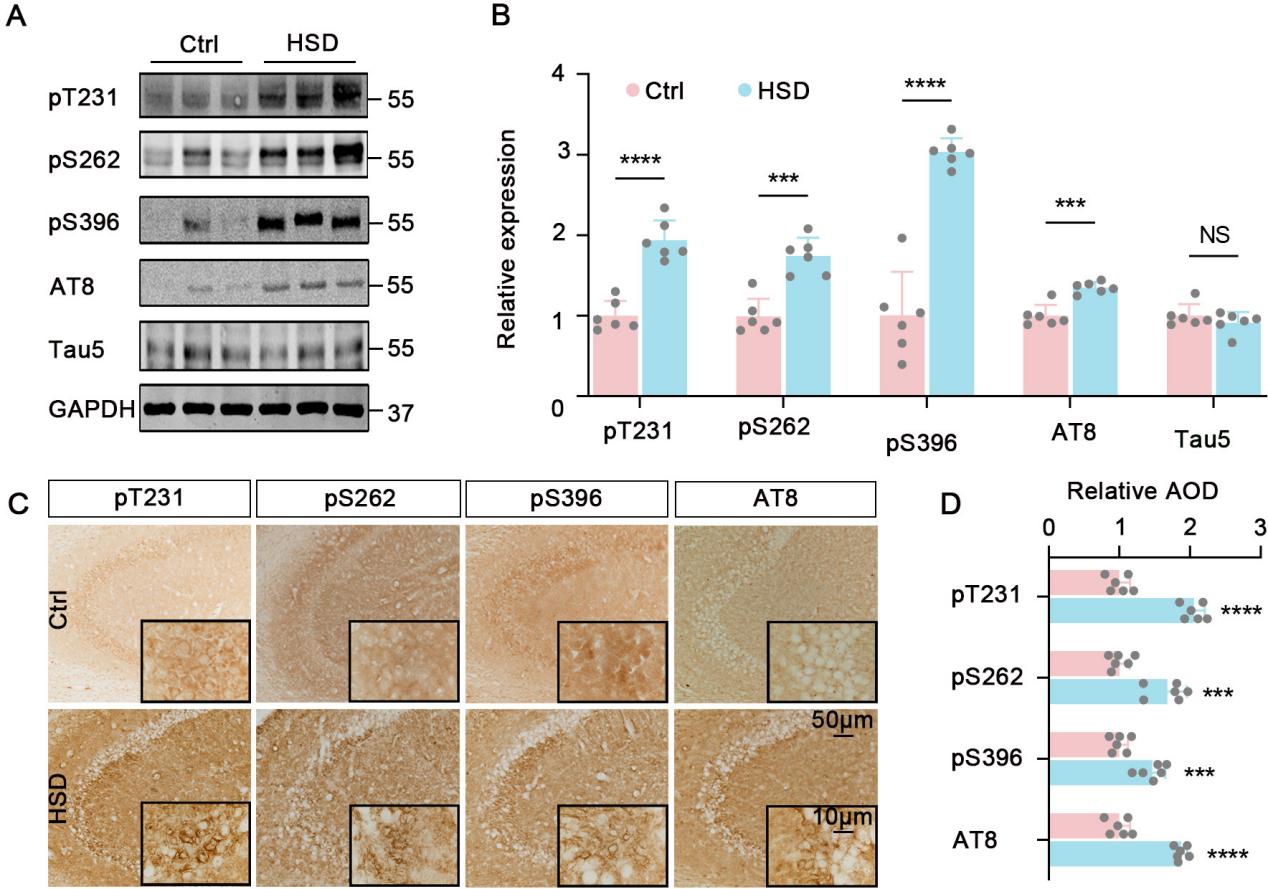


**Figure S5 Neuronal tau pathology in the HSD mice.**

(A-B) Immunoblot of the tau phosphorylation levels at different epitopes in the hippocampus of Ctrl mice and HSD mice. The representative immunoblot images (A) and the quantitative analysis (B) were shown. n = 6 mice for each group. (C) Immunocytochemistry of tau phosphorylation at different epitopes in the hippocampal CA3 region of Ctrl mice and HSD mice. (D) Releative AOD analysis of tau phosphorylation at different epitopes in the hippocampal CA3 region of Ctrl mice and HSD mice. n = 6 mice for each group.


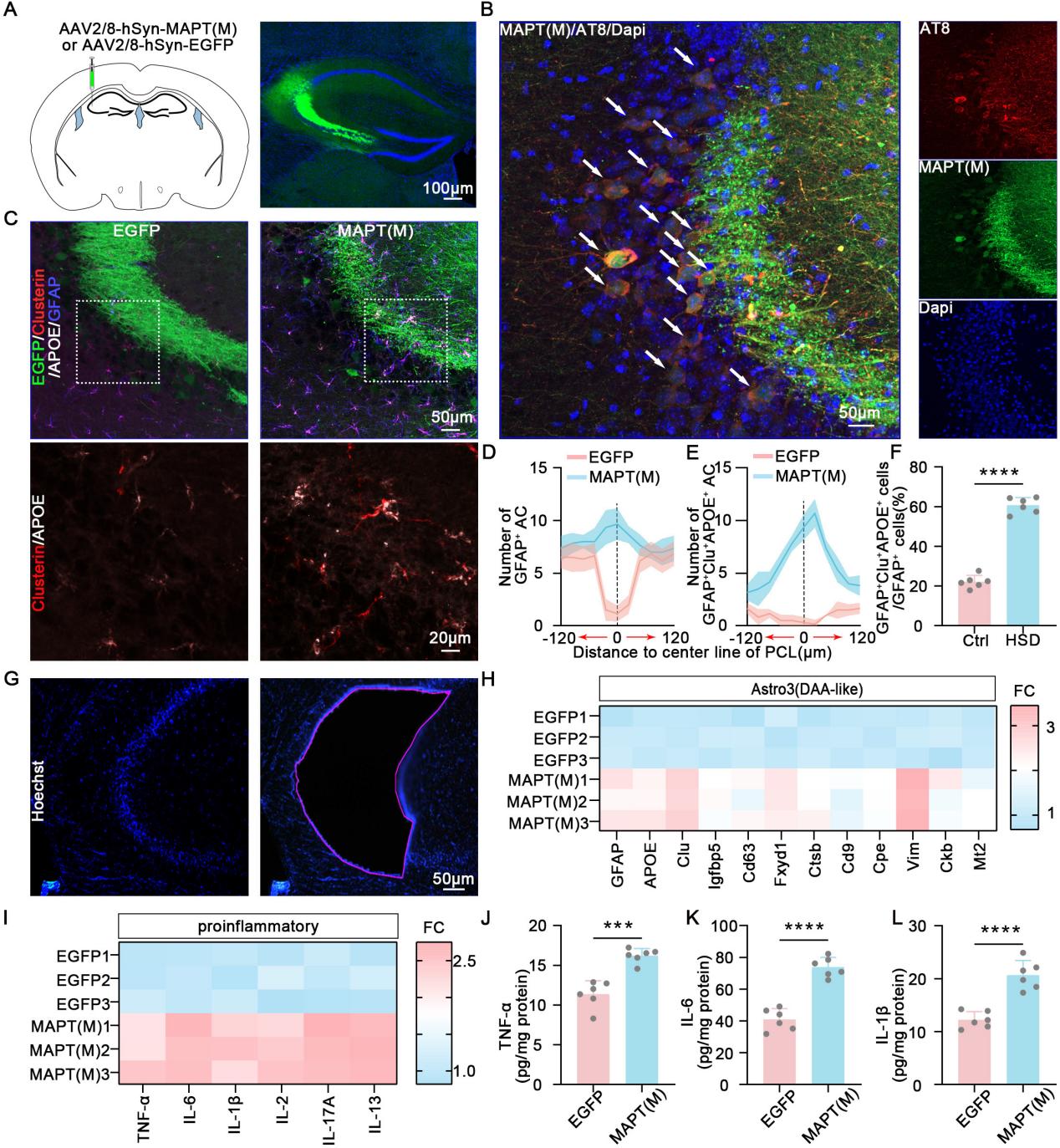


**Figure S6 Artificial induction of tau pathology triggers the DAA-like astrocyte in hippocampus.**

1. Diagram (left) and representative confocal image (right) to visualize the virus (AAV2/8-hSyn-MAPT(M)) injection site, "M" refers to mutant tau. (B) The representative immunofluorescence image for EGFP (green) and AT8 (red) in the hippocampal CA3 region of mice injected with AAV2/8-hSyn-MAPT(M). (C) The representative immunofluorescence images for EGFP (green), Clusterin (red)，APOE (white) and GFAP (blue) in the hippocampal CA3 region of mice injected with control virus and tau mutant virus. The lower panels are the enlarged images for the rectangle-labled regions in the upper panels. (D and E) The spatial distribution of GFAP^+^ astrocytes (D) and GFAP^+^Clu^+^APOE^+^ astrocytes (E) toward the center of the pyramidal cell layer. n = 6 for each group. (F) The proportion of GFAP^+^Clu^+^APOE^+^ astrocytes among GFAP^+^ astrocytes. n = 6 hippocampal slices for each group. (G) Laser capture microdissection of the hippocampal CA3 region from mice injected with control virus and tau mutant virus. Representative images before laser capture (left panel) and after laser capture (right panel) are shown. (H) qPCR to examine representative markers of DAA-like astrocytes in astrocytes sorted by magnetic beads from the hippocampus of mice injected with control virus and tau mutant virus. n = 3 mice for each group. (I) qPCR to examine the levels of TNF-α, IL-6, IL-1β, IL-2, IL-17A and IL-13 in astrocytes sorted by magnetic beads from the hippocampus of mice injected with control virus and tau mutant virus. n = 3 mice for each group. (J-L) ELISA analysis to examine the levels of TNF-α, IL-6 and IL-1β in astrocytes sorted by magnetic beads from the hippocampus of mice injected with control virus and tau mutant virus. n = 6 mice for each group.


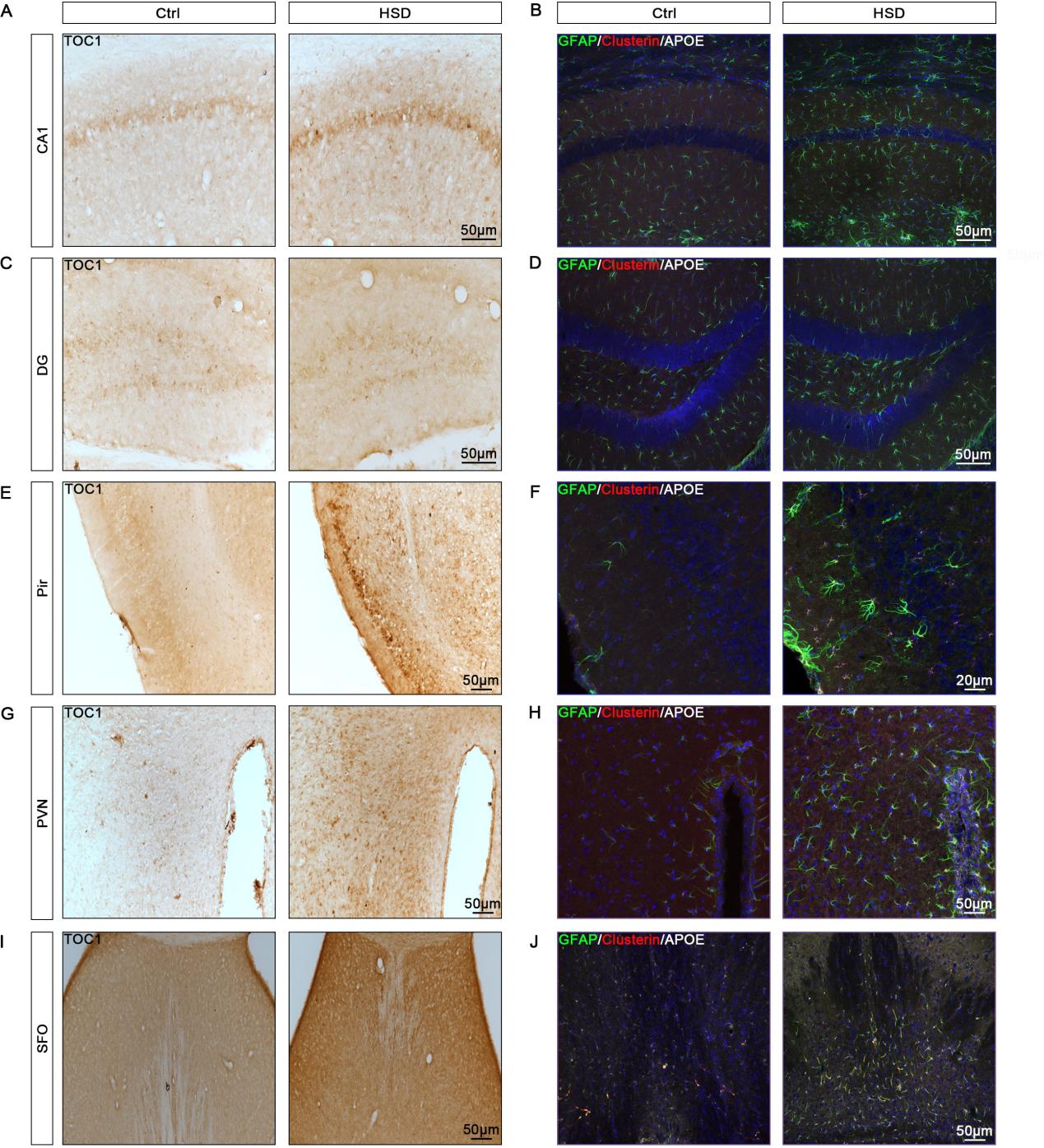


**Figure S7 The level of tau oligomer and DAA-like astrocytes in Na^+^-sensitive brain regions.**

1. Immunocytochemistry of tau oligomer in the CA1 of Ctrl mice and HSD mice. (B) Immunofluorescence for GFAP (green), Clusterin (red) and APOE (white) to label DAA-like astrocytes in the CA1 of Ctrl mice and HSD mice. Blue staining represents DAPI. (C) Immunocytochemistry of tau oligomer in the DG of Ctrl mice and HSD mice. (D) Immunofluorescence for GFAP (green), Clusterin (red) and APOE (white) to label DAA-like astrocytes in the DG of Ctrl mice and HSD mice. Blue staining represents DAPI. (E) Immunocytochemistry of tau oligomer in the piriform cortex of Ctrl mice and HSD mice. (F) Immunofluorescence for GFAP (green), Clusterin (red) and APOE (white) to label DAA-like astrocytes in the piriform cortex of Ctrl mice and HSD mice. Blue staining represents DAPI. (G) Immunocytochemistry of tau oligomer in the paraventricular nucleus of Ctrl mice and HSD mice. (H) Immunofluorescence for GFAP (green), Clusterin (red) and APOE (white) to label DAA-like astrocytes in the paraventricular nucleus of Ctrl mice and HSD mice. Blue staining represents DAPI. (I) Immunocytochemistry of tau oligomer in the subfornical organ of Ctrl mice and HSD mice. (J) Immunofluorescence for GFAP (green), Clusterin (red) and APOE (white) to label DAA-like astrocytes in the subfornical organ of Ctrl mice and HSD mice. Blue staining represents DAPI.


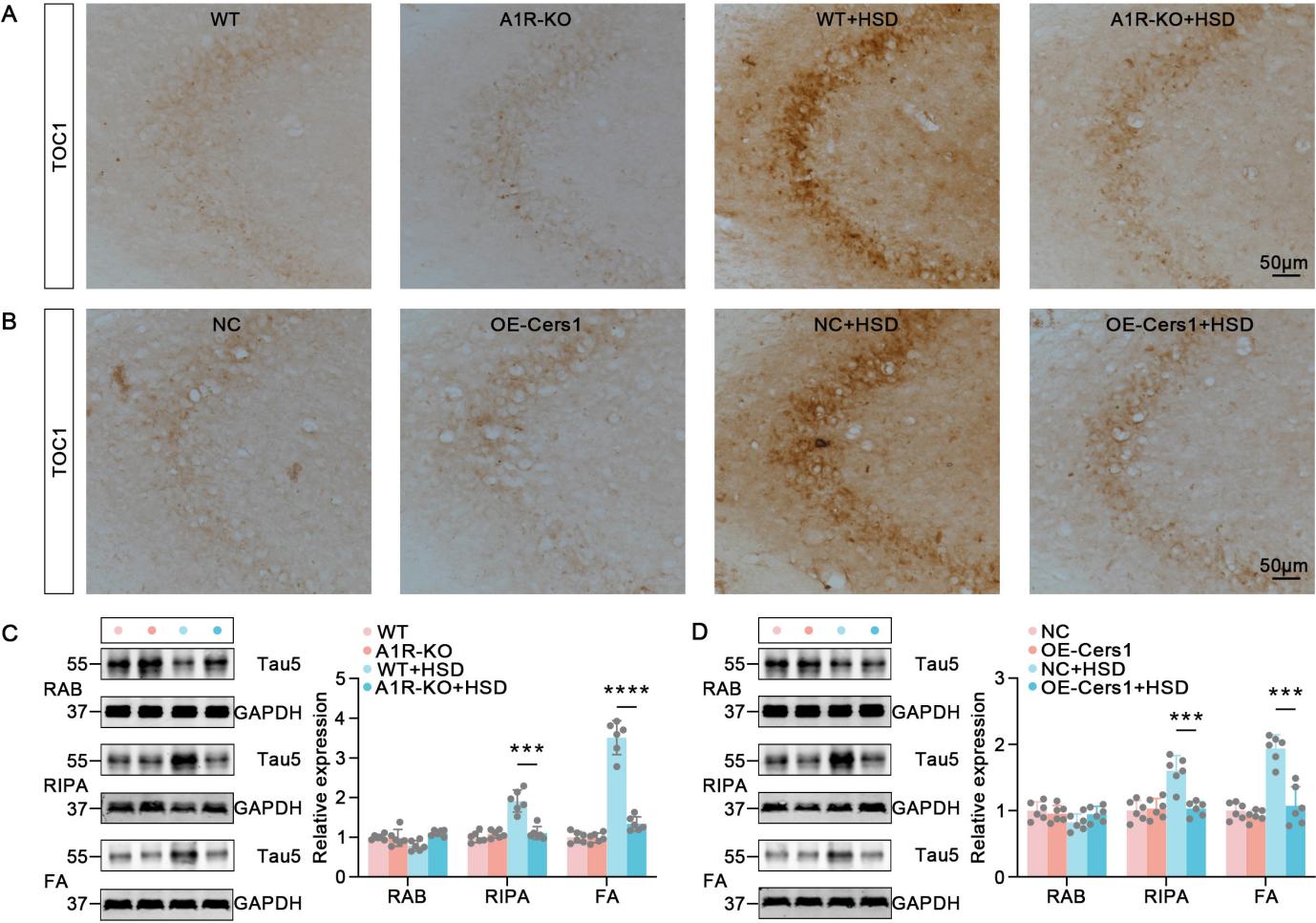


**Figure S8 Knockdown of A1R or overexpression of Cers1 reduces tau oligomer level.**

(A) Immunocytochemistry of tau oligomer in the hippocampus of WT mice, A1R-KO mice, WT + HSD mice and A1R-KO + HSD mice. (B) Immunocytochemistry of tau oligomer in the hippocampus of NC mice, OE-Cers1 mice, NC + HSD mice and OE-Cers1 + HSD mice. (C) Immunoblot of soluble, less soluble and highly insoluble tau in the hippocampus of WT mice, A1R-KO mice, WT + HSD mice and A1R-KO + HSD mice. n = 6 mice for each group. (D) Immunoblot of soluble, less soluble and highly insoluble tau in the hippocampus of NC mice, OE-Cers1 mice, NC + HSD mice and OE-Cers1 + HSD mice. n = 6 mice for each group.


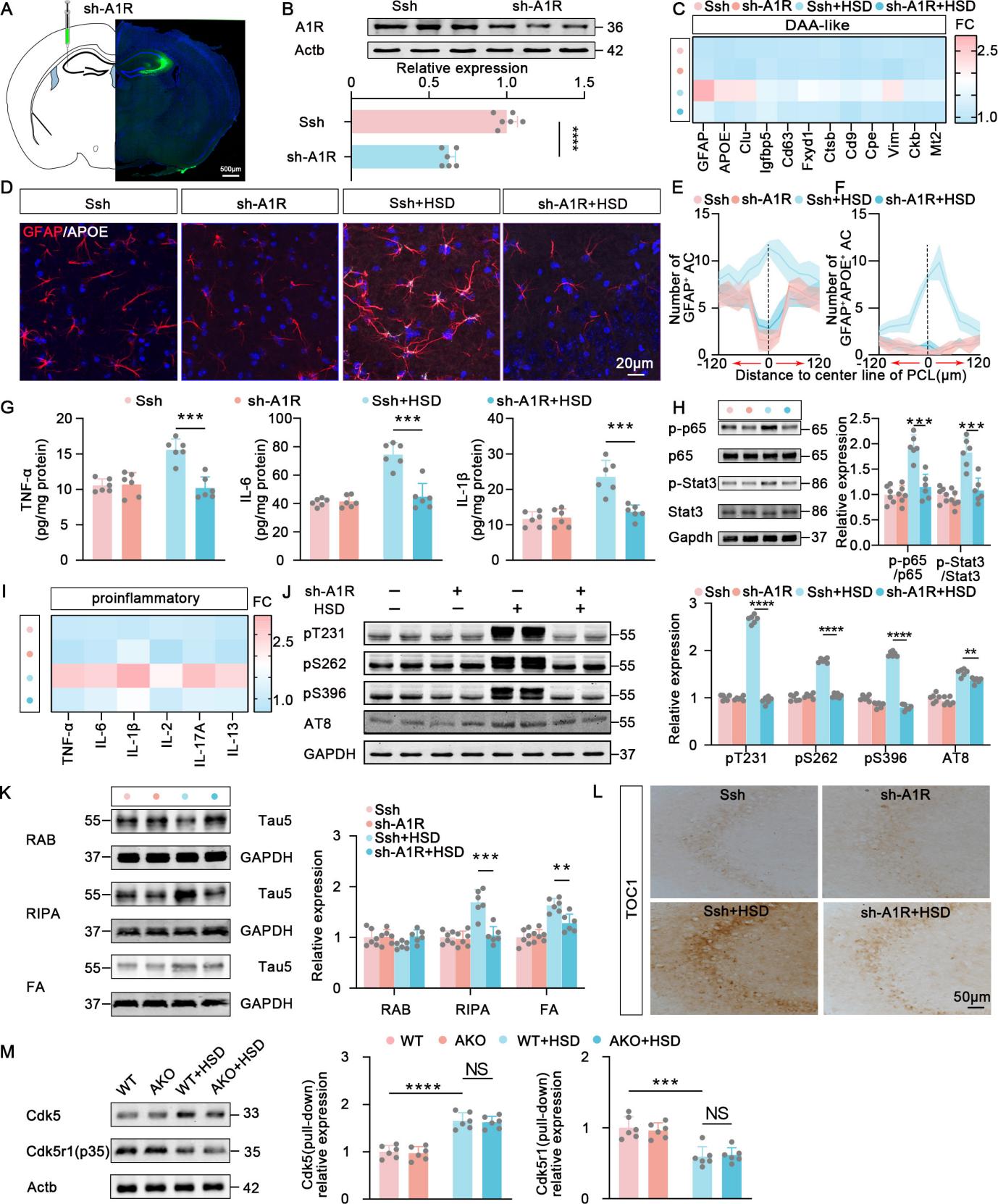


**Figure S9 Sliencing of neuronal A1R reduces DAA-like astrocytes and ameliorates tau pathology in HSD mice.**

1. Diagram (left) and representative confocal image (right) of Lenti-hSyn1-miR30-shA1R-P2A-EGFP (sh-A1R) lentivirus infection. Blue staining represents DAPI. (B) The representative immunoblot of A1R and Actin (upper), the quantitative analysis (lower) in the hippocampus of mice injected with sh-A1R virus or scrambled shRNA (Ssh) virus. n = 6 mice for each group. (C) qPCR to examine the representative markers of DAA-like astrocytes in astrocytes sorted by magnetic beads from the hippocampus of Ssh mice, sh-A1R mice, Ssh+HSD mice and sh-A1R + HSD mice. n = 6 mice for each group. (D) The representative immunofluorescence images for GFAP (red) and APOE (white) in the hippocampal CA3 region of Ssh mice, sh-A1R mice, Ssh+HSD mice and sh-A1R + HSD mice. Blue staining represents DAPI. (E and F) The spatial distribution of GFAP^+^ astrocytes (E) and GFAP^+^Clu^+^APOE^+^ astrocytes (F) toward the center of the pyramidal cell layer. n = 6 hippocampal slices for each group. (G) ELISA analysis to examine the levels of `astrocytes sorted by magnetic beads from the hippocampus of Ssh mice, sh-A1R mice, Ssh+HSD mice and sh-A1R + HSD mice. n = 6 mice for each group. (H) Immunoblot of total and phosphorylation levels NF-κB and STAT3 (left) and the quantitative analysis (right) in the hippocampus of Ssh mice, sh-A1R mice, Ssh+HSD mice and sh-A1R + HSD mice. n = 6 mice for each group. (I) qPCR to examine the mRNA levels of TNF-α, IL-6, IL-1β, IL-2, IL-17A and IL-13 in astrocytes sorted by magnetic beads from the hippocampus of Ssh mice, sh-A1R mice, Ssh+HSD mice and sh-A1R + HSD mice. n = 6 mice for each group. (J) Immunoblot of the tau phosphorylation levels at different epitopes in the hippocampus of Ssh mice, sh-A1R mice, Ssh+HSD mice and sh-A1R + HSD mice. n = 6 mice for each group. (K) Immunoblot of soluble, less soluble and highly insoluble tau in the hippocampus of Ssh mice, sh-A1R mice, Ssh+HSD mice and sh-A1R + HSD mice. n = 6 mice for each group. (L) Immunocytochemistry of tau oligomer in the hippocampus of Ssh mice, sh-A1R mice, Ssh+HSD mice and sh-A1R + HSD mice. (M) Immunoblot of Cdk5 (pull-down) and Cdk5r1 (pull-down) in the hippocampus of WT mice, A1R-KO mice, WT + HSD mice and A1R-KO + HSD mice. n = 6 mice for each group.


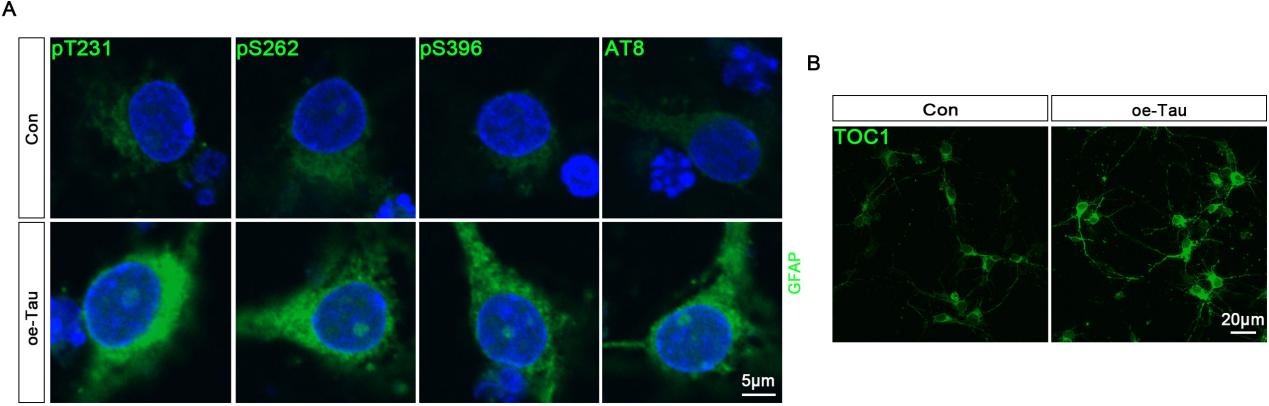


**Figure S10 Verification of tau pathology in primary neurons.**

1. The representative immunofluorescence image for tau phosphorylation in the primary neurons treated with oe-Tau and Con. Blue staining represents DAPI. (B) The representative immunofluorescence image for tau oligomer in the primary neurons treated with oe-Tau and Con.


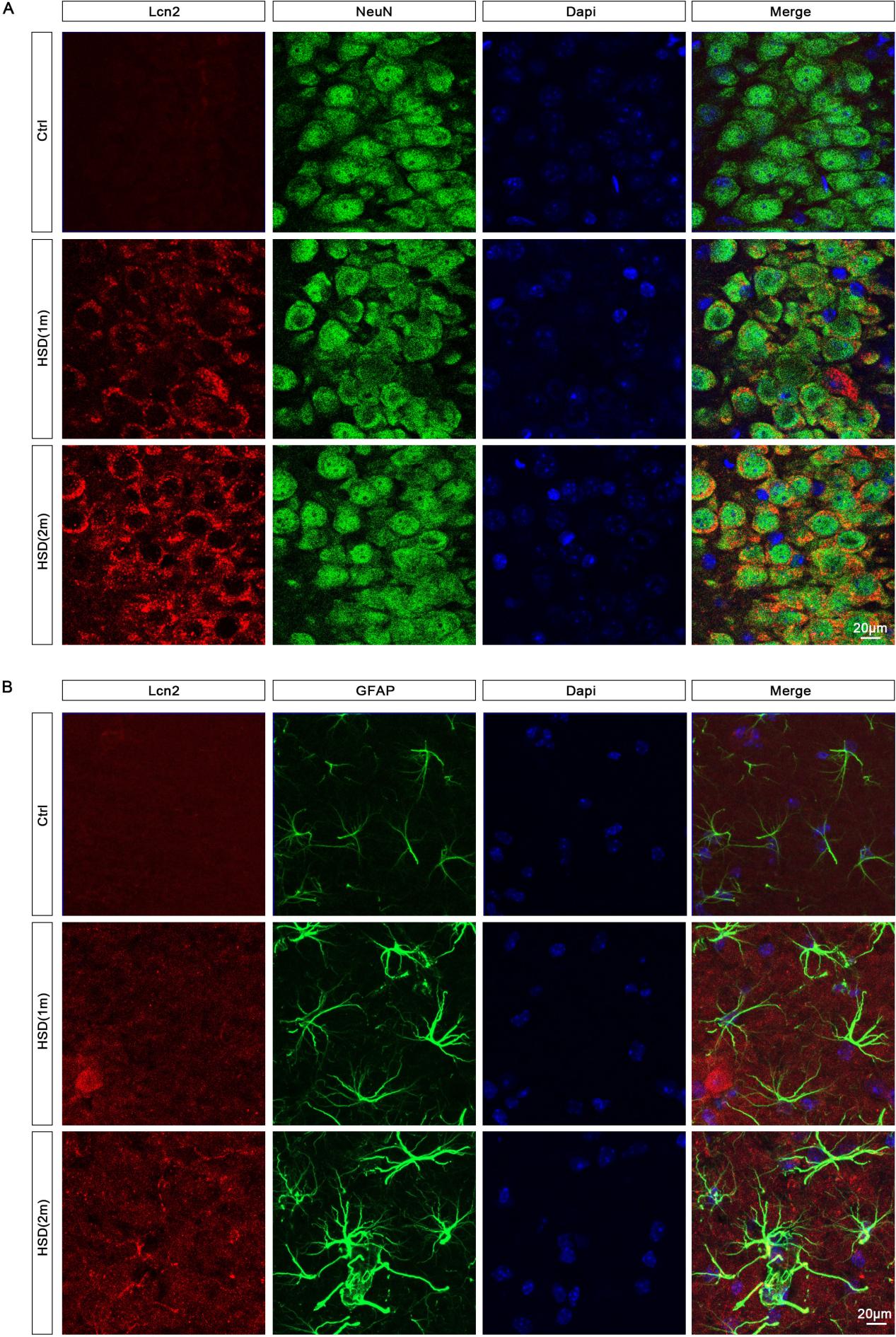


**Figure S11 Lcn2 is primarily released by neurons in one-month HSD mice.**

1. The representative immunofluorescence images for NeuN (green), Lcn2 (red) and Dapi (blue) in the hippocampus of Ctrl mice, one-month HSD mice and two-month HSD mice. (B) The representative immunofluorescence images for GFAP (green), Lcn2 (red) and Dapi (blue) in the hippocampus of Ctrl mice, one-month HSD mice and two-month HSD mice.


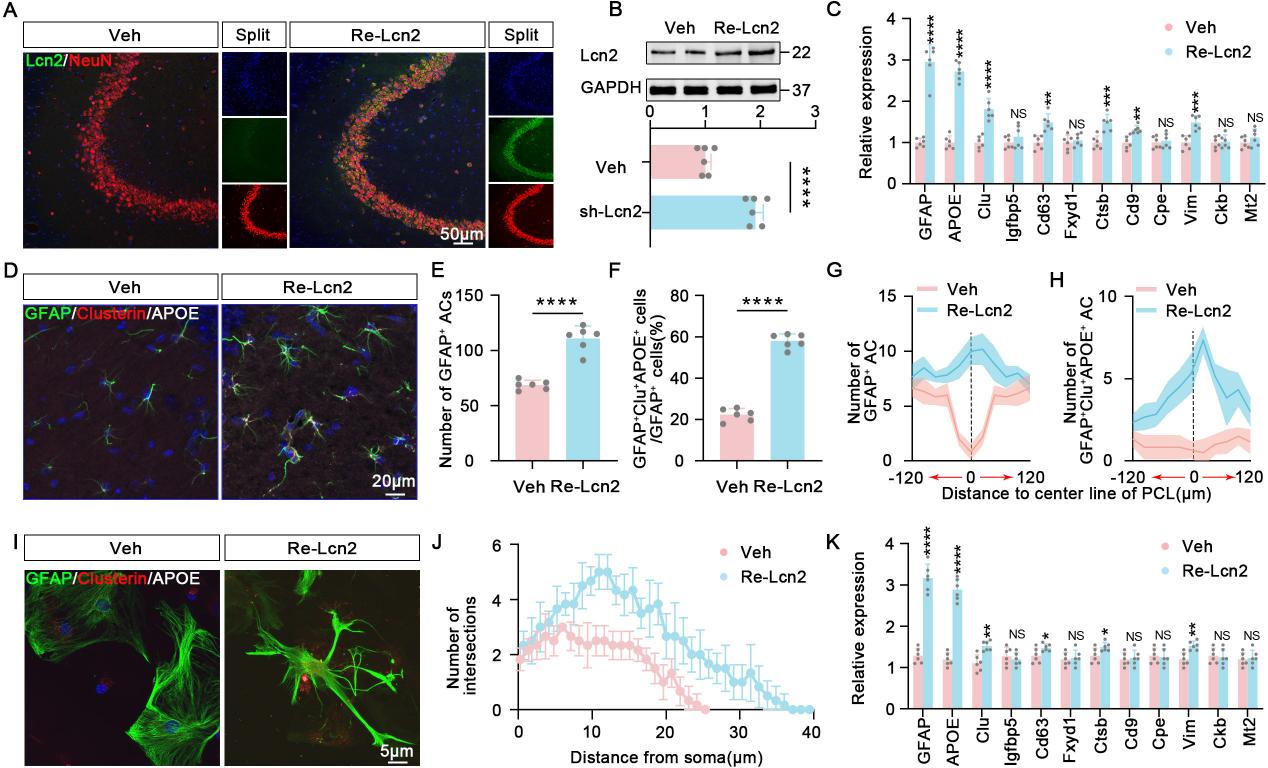


**Figure S12 Administration of** **Lcn2 recom****binant protein partially induces DAA-like astroglial activation both *in vivo* and *in vitro*.**

1. The representative immunofluorescence images for Lcn2 (green) and NeuN (red) in the hippocampal CA3 region of mice injected with recombinant Lcn2 (Re-Lcn2) or vehicle (Veh). Blue staining represents DAPI. (B) Immunoblots of Lcn2 and GAPDH in the hippocampus of mice injected with Re-Lcn2 or Veh. n = 6 mice for each group. (C) qPCR to examine the representative markers of DAA-like astrocytes in astrocytes sorted by magnetic beads from the hippocampus of mice injected with Re-Lcn2 or Veh. n = 6 mice for each group. (D) The representative immunofluorescence images for GFAP (green), Clusterin (red) and APOE (white) in the hippocampal CA3 region of mice injected with Re-Lcn2 or Veh. Blue staining represents DAPI. (E) Number of GFAP^+^ astroccytes in the hippocampal CA3 region of mice injected with Re-Lcn2 or Veh. n = 6 hippocampal slices for each group. (F) The proportion of GFAP^+^Clu^+^APOE^+^ astrocytes in total GFAP^+^ astrocytes. n = 6 hippocampal slices for each group. (G and H) The spatial distribution of GFAP^+^ astrocytes (G) and GFAP^+^Clu^+^APOE^+^ astrocytes (H) toward the center of the pyramidal cell layer. n = 6 hippocampal slices for each group. (I) The representative immunofluorescence images for GFAP (green), Clusterin (red) and APOE (white) in the primary astrocytes treated with Veh or Re-Lcn2. Blue staining represents DAPI. (J) Number of intersections of primary astrocytes treated with Veh or Re-Lcn2. n = 6 independent experiments for each group. (K) qPCR to examine the representative markers of DAA-like astrocytes in primary astrocytes treated with Veh or Re-Lcn2. n = 6 independent experiments for each group.

**Table S1 Reagents used in this study.**

| Antibodies | | | |
| --- | --- | --- | --- |
| A1R (rabbit polyclonal) | 1:500 for WB | Proteintech | 55026-1-AP |
| A1R (rabbit polyclonal) | 1:200 for IF | Abcam | ab82477 |
| NeuN (rabbit polyclonal) | 1:500 for IF | Abcam | ab104225 |
| NeuN (mouse polyclonal) | 1:500 for IF | Abcam | ab104224 |
| GFAP (mouse monoclonal) | 1:500 for IF | CST | 3670 |
| Ibal (chicken polyclonal) | 1:500 for IF | Abcam | ab318302 |
| Ibal (rabbit polyclonal) | 1:500 for IF | Wako | 019-19741 |
| MAP2 (rabbit polyclonal) | 1:1000 for IF | Proteintech | 17490-1-AP |
| Tau5 (mouse monoclonal) | 1:1000 for WB,  1:200 for IF | Invitrogen | AHB0042 |
| pT231 (mouse monoclonal) | 1:1000 for WB | Signalway | #11110 |
| pS262 (mouse monoclonal) | 1:1000 for WB | Signalway | #11111 |
| pS396 (mouse monoclonal) | 1:1000 for WB | Signalway | #11102 |
| AT8 (mouse monoclonal) | 1:1000 for WB | Signalway | MN1020 |
| Lcn2 (rabbit polyclonal) | 1:1000 for WB,  1:200 for IF | Abcam | ab63929 |
| p65 (rabbit polyclonal) | 1:1000 for WB | Abcam | ab32536 |
| p65 (S536) (rabbit polyclonal) | 1:1000 for WB | Abcam | ab239882 |
| STAT3 (rabbit polyclonal) | 1:1000 for WB | Abcam | ab68153 |
| STAT3 (Y705) (rabbit polyclonal) | 1:1000 for WB | Abcam | ab76315 |
| Cers1 (rabbit polyclonal) | 1:1000 for WB | Abcam | ab198799 |
| 24p3R (rabbit polyclonal) | 1:1000 for WB | Abcam | ab237539 |
| Clusterin (Goat polyclonal) | 1:200 for IF | thermofisher | PA5-46931 |
| APOE (rabbit polyclonal) | 1:200 for IF | CST | 49285 |
| S1pr1 (rabbit polyclonal) | 1:200 for IF | Proteintech | 55133-1-AP |
| Tspan7 (rabbit polyclonal) | 1:200 for IF | ABclonal | A6417 |
| MBP (rabbit polyclonal) | 1:500 for IF | Abcam | ab209328 |
| TNF-α (rabbit polyclonal) | 1:200 for IF | Abcam | ab183218 |
| β-actin (mouse monoclonal) | 1:2000 for WB | Proteintech | 60008-1-Ig |
| GAPDH (mouse monoclonal) | 1:2000 for WB | Proteintech | 60004-1-Ig |
| Goat anti-Mouse IgG Secondary Antibody, Alexa Fluor 488 | 1:500 for IF | Invitrogen | A-11001 |
| Goat anti-Rabbit IgG Secondary Antibody, Alexa Fluor 488 | 1:500 for IF | Invitrogen | A-11008 |
| Goat anti-Mouse IgG Secondary Antibody, Alexa Fluor 546 | 1:500 for IF | Invitrogen | A-11003 |
| Goat anti-Rabit IgG Secondary Antibody, Alexa Fluor 546 | 1:500 for IF | Invitrogen | A-11010 |
| Chicken Anti-Goat IgG Secondary Antibody, Alexa Fluor 647 | 1:500 for IF | Invitrogen | A-21469 |
| Goat Anti-Mouse IgG Secondary Antibody, Alexa Fluor 647 | 1:500 for IF | Abcam | ab150115 |
| IRDye 800CW Goat anti-Mouse IgG | 1:10000 for WB | LI-COR Biosciences | 926-32210 |
| IRDye 800CW Goat anti-Rabbit IgG | 1:10000 for WB | LI-COR Biosciences | 926-32211 |

| Reagents and recombinant proteins | | |
| --- | --- | --- |
| DMEM/F12 | Invitrogen | 11320033 |
| Neurobasal-A ([-] L-glutamine) | Life Technologies | 10888-022 |
| B-27 supplement | Life Technologies | 12587010 |
| GlutaMAX Supplement | Life Technologies | 35050061 |
| Penicillin-streptomycin 50X | Life Technologies | 15140122 |
| 0.25% Trypsin (1X) | GIBCO | 15050-065 |
| Recombinant mouse Lcn2 | Abcam | ab282399 |

| Virus strains | | |
| --- | --- | --- |
| Lenti-hSyn1-miR30-shA1R-P2A-EGFP | Taitool Bioscience | N/A |
| rAAV2/9-hSyn1-miR30-shLcn2-P2A-mCherry | Taitool Bioscience | N/A |
| Lenti-U6-shScramble | Taitool Bioscience | N/A |
| Lenti-U6-shTau | Taitool Bioscience | N/A |
| AAV2/8-hSyn-Cers1-EGFP | Taitool Bioscience | N/A |
| AAV2/8-hSyn-MAPT(M)-EGFP | Taitool Bioscience | N/A |
| AAV5-GfaABC1D-sh-24p3R-mCherry | Taitool Bioscience | N/A |

| Critical commercial assays | | |
| --- | --- | --- |
| Mouse TNF-α ELISA Kit | Elabscience | E-EL-M3063 |
| Mouse IL-6 ELISA Kit | Elabscience | E-EL-M0044 |
| Mouse IL-1β ELISA Kit | Elabscience | E-EL-M0037 |
| Mouse Cers1 ELISA Kit | mlbio | ml062988 |
| Mouse Ceramide ELISA Kit | mlbio | ml037499 |
| PP2A activity assay Kit | halingbio | HL50042.4 |
| Mouse Asph ELISA Kit | mlbio | YJ988717 |
| Mouse Lcn2 ELISA Kit | Elabscience | E-EL-M0828 |
| Mouse Adm ELISA Kit | Elabscience | E-EL-M2421 |
| Mouse Mfap5 ELISA Kit | mlbio | YJ920150 |
| Mouse Tnn ELISA Kit | mlbio | YJ969052 |
| Mouse Ndufb10 ELISA Kit | mlbio | YJ970025 |
| Mouse Sdc1 ELISA Kit | mlbio | ml037982 |
| Mouse Plcb1 ELISA Kit | mlbio | YJ974141 |
| BCA Protein Assay Kit | Thermo SCIENTIFIC | 23227 |

| Experimental models: organisms/strains | | |
| --- | --- | --- |
| Mouse: C57BL/6J | The Jackson Laboratory | RRID: MMRRC_034830-JAX |
| Mouse: A1R KO | Zhou *et al.*, 2018 | RRID: IMSR_JAX:014161 |
| Mouse: Tau KO | Zheng *et al.* 2021 | N/A |

| Software and algorithms | | |
| --- | --- | --- |
| ImageJ | NIH | https://imagej.net/software/imagej/ |
| Fiji |  | https://imagej.net/software/fiji/ |
| GraphPad Prism 8 | GraphPad Software Inc | https://www.graphpad.com/scientific-software/prism/ |
| Imaris 9.0 | Bitplane | https://imaris.oxinst.com/newrelease |

**Table S2 The sequence of primers and shRNAs used in this study.**

| **gene** | **sequence(5'-3')** |
| --- | --- |
| GFAP qPCR F | CCTTCTGACACGGATTTGGT |
| GFAP qPCR R | TAAGCTAGCCCTGGACATCG |
| Vimetin qPCR F | AGCGTGGCTGCCAAGAACCTC |
| Vimetin qPCR R | GCAGGGCATCGTTGTTCCGGT |
| Clusterin qPCR F | GCTGCTGATCTGGGACAATG |
| Clusterin qPCR R | ACCTACTCCCTTGAGTGGACA |
| Igfbp5 qPCR F | TGCCGCAGACACATGGAA |
| Igfbp5 qPCR R | TGCGTGGGCTGGCTTT |
| Cd63 qPCR F | TCATCCAAACGTGTATCCTTCTG |
| Cd63 qPCR R | CTTGTGCTCGGACCCTTTTCT |
| Fxyd1 qPCR F | TCCATGGCCAGTGCAGAA |
| Fxyd1 qPCR R | ATGAAGAGGATCCCAGCGATA |
| Ctsb qPCR F | AGGTTCGGTCAGAAATGGCTT |
| Ctsb qPCR R | ATCCTTCTTTCTTGCCTGCTG |
| Ctsb qPCR F | TGGGGCTATACCCACAAGGA |
| Ctsb qPCR R | GCTTTGAGTGTTTCCCGCTG |
| Cd9 qPCR F | TGGGGCTATACCCACAAGGA |
| Cd9 qPCR R | GCTTTGAGTGTTTCCCGCTG |
| Cpe qPCR F | TTCTGTGACTCCAGCCTGTG |
| Cpe qPCR R | GGAGCATGGTCCTCTCTCAG |
| APOE qPCR F | GATCCGATCCCCTGCTCAGA |
| APOE qPCR R | TCTGTCACCTCCGGCTCTCC |
| Ckb qPCR F | GCTCATTGACGACCACTTCC |
| Ckb qPCR R | AGTTTCACTCCGTCCACCAC |
| Mt2 qPCR F | ACTTCGTGGGGAACCTGCTTG |
| Mt2 qPCR R | TTGAAGACAGAGCCAATGACACT |
| S1pr1 qPCR F | ATGGTGTCCACTAGCATCCC |
| S1pr1 qPCR R | CGATGTTCAACTTGCCTGTGTAG |
| Ndrg2 qPCR F | CGCATCCTCCTGGACCAGGGAC |
| Ndrg2 qPCR R | CATCATGGTAGGTGAATATCGC |
| Gjb6 qPCR F | CCCAATCTCGTGGACTGCTTC |
| Gjb6 qPCR R | GTAACACAACTCGGCCACATTGA |
| Glud1 qPCR F | CGACGCCACTACAGCGAAG |
| Glud1 qPCR R | GCTCAACACATGGTTGCAGG |
| Vegfa qPCR F | CCACGTCAGAGAGCAACATCA |
| Vegfa qPCR R | TCATTCTCTCTATGTGCTGGCTTT |
| Tspan7 qPCR F | ACCAGTTTTATGGAGACTAACATGG |
| Tspan7 qPCR R | AGCAGCATGCCAATCAACT |
| Ttyh1 qPCR F | GACCCTTTCAACCCTCAGGAAT |
| Ttyh1 qPCR R | GCTTGTAAAATAAAGCCTTCCCTTTT |
| Gldc qPCR F | AGCATTGATGAGCTCATCGAG |
| Gldc qPCR R | TCCAGCAGGGAAGCGTTGGC |
| Gpm6b qPCR F | ACACCAGTGACCATGCCTTTCC |
| Gpm6b qPCR R | GACATCCACACAGATCTGCTCCA |
| TNF-α qPCR F | TCTTCTCATTCCTGCTTGTGG |
| TNF-α qPCR R | ATGAGAGGGAGGCCATTTG |
| IL-6 qPCR F | CAAAGCCAGAGTCCTTCAGAG |
| IL-6 qPCR R | AGCATTGGAAATTGGGGTAG |
| IL-1β qPCR F | CAGCCTTATTTCGGGAGTCTATTC |
| IL-1β qPCR R | TATCCCTTTGTTAACCCATCTGTA |
| IL-1β qPCR F | GCATGCAGCTCGCATCCTGTG |
| IL-1β qPCR R | TTCTGTGGCCTGCTTGGGCA |
| IL-17A qPCR F | CTGGAGGATAACACTGTGAGAGT |
| IL-17A qPCR R | TGCTGAATGGCGACGGAGTTC |
| IL-13 qPCR F | CAGCTCCCTGGTTCTCTCAC |
| IL-13 qPCR R | ACACTCCATACCATGCTGCC |
| IL-4 qPCR F | AGGAGCCATATCCACGGATG |
| IL-4 qPCR R | ACAGACGAGCTCACTCTCTG |
| IL-10 qPCR F | TCACTCTTCACCTGCTCCAC |
| IL-10 qPCR R | CTATGCTGCCTGCTCTTACTC |
| TGFb qPCR F | TGCGCTTGCAGAGATTAAAA |
| TGFb qPCR R | CTGCCGTACAACTCCAGTGA |
| IL-11 qPCR F | AGGCGAGACATCAAGAGCTG |
| IL-11 qPCR R | GCAGGTGGTCCTTCCCTAA |
| Cers1 qPCR F | AGAAGCAGGTAGATTTCTGTGAGTTC |
| Cers1 qPCR R | GAAACAAAGAGAAAATGTTTCAAAATG |
| Smco1 qPCR F | GTCTTCCGACTTTAGCCTCTG |
| Smco1 qPCR R | GCATCCGTCTTACATTAGCG |
| Chmp2a qPCR F | AAGGCCAGATGGATGCTGT |
| Chmp2a qPCR R | CCGCATCAACACAAACTTGC |
| Lcn2 qPCR F | CTGTCCCAATCGACCAGTGT |
| Lcn2 qPCR R | CCAGCTCCCTCAATGGTGTT |
| Pon1 qPCR F | CACCCGTCTCGATTCCTTTA |
| Pon1 qPCR R | CAGCCTGTCCATCTGTCTCA |
| Sap25 qPCR F | GGAGGCTGTGATCCTGGTTG |
| Sap25 qPCR R | CCGAGTGTAGACGACATAGAG |
| Hp qPCR F | AACTCCCCGAATGTGAGGCA |
| Hp qPCR R | CGTGGCGGGAGATCATCTTG |
| Cndp1 qPCR F | GAAGAATACCGGAATAGCAG |
| Cndp1 qPCR R | CGGCCAGGTATGACTGTT |
| Tc2n qPCR F | TGGCTGTACTGAGGATTATTTGC |
| Tc2n qPCR R | TGTGAAGGAGTTTCTTGTGTCC |
| Sh3rf2 qPCR F | cctctcttcctatgcgctca |
| Sh3rf2 qPCR R | tgcagctgttgttgtgactc |
| GAPDH qPCR F | TGTTTCCTCGTCCCGTAG |
| GAPDH qPCR R | CAATCTCCACTTTGCCACT |
| sh-A1R | CCGGGAAGGTTATCAGCATGGAGTACTCGAGTACTCCATGCTGATAACCTTCTTTTTTGGTACC |
| sh-hTau | CCGGGTGTGGCTCATTAGGCAACATCTCGAG-ATGTTGCCTAATGAGCCACAC-TTTTTTGGTACC |
| sh-Lcn2 | TGCTGTCAAGTTCTGAGTTGAGTCCTGTTTTGGCCACTGACTGACAGGACTCATCAGAACTTGA |

**Table S3 Statistic analysis data for all the figures.**

| Figure | Statistic method | Number(n) | Statistic results | Value |
| --- | --- | --- | --- | --- |
| 1C | Two-tailed t test | 6, 6 | *p* = 0.0002 | t=5.748, df=10 |
| 1D | Two-tailed t test | 79, 118 | *p* < 0.0001 | t=17.85, df=196 |
| 1E | Two-tailed t test | 69, 102 | *p* <0.0001 | t=10.83, df=168 |
| 1J | Two-tailed t test | 6, 6 | *p* <0.0001 | t=11.45, df=10 |
| 2B upper | Two-tailed t test | 6, 6 | *p* = 0.5882 | t=0.5593, df=10 |
| 2B lower | Two-tailed t test | 6, 6 | *p* = 0.8336 | t=0.2157, df=10 |
| 2F | Two-tailed t test | 6, 6 | *p* = 0.0001 | t=6.034, df=10 |
| 2J | Two-tailed t test | 6, 6 | *p* = 0.0439 | t=2.083, df=10 |
| 3B lower | Two-tailed t test | 6, 6 | *p* < 0.0001 | t=8.013, df=10 |
| 3I | One-way ANOVA with Tukey's multiple comparisons test | 6, 6, 6, 6 | *p* < 0.0001 | F (3, 20) = 264.6 |
| 3J | Linear regression | 12 | *p* < 0.0001 | R^2^ = 0.8478 |
| 3L | One-way ANOVA with Tukey's multiple comparisons test | 6, 6, 6, 6 | *p* = 0.0041 | F (3, 20) = 8.395 |
| 3M | One-way ANOVA with Tukey's multiple comparisons test | 6, 6, 6, 6 | *p* = 0.0031 | F (3, 20) = 15.23 |
| 3N | One-way ANOVA with Tukey's multiple comparisons test | 6, 6, 6, 6 | *p* = 0.0113 | F (3, 20) = 19.02 |
| 3P p-p65/p65 | One-way ANOVA with Tukey's multiple comparisons test | 6, 6, 6, 6 | *p* = 0.0013 | F (3, 20) = 43.77 |
| 3P p-Stat3/Stat3 | One-way ANOVA with Tukey's multiple comparisons test | 6, 6, 6, 6 | *p* < 0.0001 | F (3, 20) = 28.67 |
| 4B upper | Two-tailed t test | 6, 6 | *p* < 0.0001 | t=16.61, df=10 |
| 4B lower | Two-tailed t test | 6, 6 | *p* < 0.0001 | t=12.92, df=48 |
| 4D Hp | Two-tailed t test | 6, 6 | *p* < 0.0001 | t=14.25, df=10 |
| 4D Primary | Two-tailed t test | 6, 6 | *p* < 0.0001 | t=9.138, df=10 |
| 4F left | One-way ANOVA with Tukey's multiple comparisons test | 6, 6, 6, 6 | *p* < 0.0001 | F (3, 20) = 76.33 |
| 4F right | One-way ANOVA with Tukey's multiple comparisons test | 6, 6, 6, 6 | *p* < 0.0001 | F (3, 20) = 82.09 |
| 4H | One-way ANOVA with Tukey's multiple comparisons test | 6, 6, 6, 6 | *p* < 0.0001 | F (3, 20) = 87.65 |
| 4J | Two-tailed t test | 6, 6 | *p* < 0.0001 | t=14.49, df=10 |
| 4O left | One-way ANOVA with Tukey's multiple comparisons test | 6, 6, 6, 6 | *p* = 0.0060 | F (3, 20) = 13.47 |
| 4O middle | One-way ANOVA with Tukey's multiple comparisons test | 6, 6, 6, 6 | *p* = 0.0004 | F (3, 20) = 31.19 |
| 4O right | One-way ANOVA with Tukey's multiple comparisons test | 6, 6, 6, 6 | *p* = 0.0027 | F (3, 20) = 13.54 |
| 4P right p-p65/p65 | One-way ANOVA with Tukey's multiple comparisons test | 6, 6, 6, 6 | *p* = 0.0005 | F (3, 20) = 37.32 |
| 4P right p-Stat3/Stat3 | One-way ANOVA with Tukey's multiple comparisons test | 6, 6, 6, 6 | *p* = 0.0003 | F (3, 20) = 24.16 |
| 4R right pT231 | One-way ANOVA with Tukey's multiple comparisons test | 6, 6, 6, 6 | *p* < 0.0001 | F (3, 20) = 101.7 |
| 4R right pS262 | One-way ANOVA with Tukey's multiple comparisons test | 6, 6, 6, 6 | *p* < 0.0001 | F (3, 20) = 94.83 |
| 4R right pS396 | One-way ANOVA with Tukey's multiple comparisons test | 6, 6, 6, 6 | *p* < 0.0001 | F (3, 20) = 286.9 |
| 4R right AT8 | One-way ANOVA with Tukey's multiple comparisons test | 6, 6, 6, 6 | *p* < 0.0001 | F (3, 20) = 128.0 |
| 5E  AKO *vs* WT | One-way ANOVA with Tukey's multiple comparisons test | 6, 6, 6, 6 | *p* = 0.0068 | F (3, 20) = 64.74 |
| 5E  WT+HSD *vs* WT | One-way ANOVA with Tukey's multiple comparisons test | 6, 6, 6, 6 | *p* < 0.0001 | F (3, 20) = 64.74 |
| 5E  AKO+HSD *vs* WT+HSD | One-way ANOVA with Tukey's multiple comparisons test | 6, 6, 6, 6 | *p* < 0.0001 | F (3, 20) = 64.74 |
| 5F  AKO *vs* WT | One-way ANOVA with Tukey's multiple comparisons test | 6, 6, 6, 6 | *p* = 0.0002 | F (3, 20) = 41.96 |
| 5F  WT+HSD *vs* WT | One-way ANOVA with Tukey's multiple comparisons test | 6, 6, 6, 6 | *p* = 0.0003 | F (3, 20) = 41.96 |
| 5F  WT+HSD *vs* WT | One-way ANOVA with Tukey's multiple 6, 6, 6, 6comparisons test | 6, 6, 6, 6 | *p* < 0.0001 | F (3, 20) = 41.96 |
| 5G  AKO *vs* WT | One-way ANOVA with Tukey's multiple comparisons test | 6, 6, 6, 6 | *p* = 0.0003 | F (3, 20) = 59.24 |
| 5G  WT+HSD *vs* WT | One-way ANOVA with Tukey's multiple comparisons test | 6, 6, 6, 6 | *p* < 0.0001 | F (3, 20) = 59.24 |
| 5G  AKO+HSD *vs* WT+HSD | One-way ANOVA with Tukey's multiple comparisons test | 6, 6, 6, 6 | *p* < 0.0001 | F (3, 20) = 59.24 |
| 5H  AKO *vs* WT | One-way ANOVA with Tukey's multiple comparisons test | 6, 6, 6, 6 | *p* = 0.0008 | F (3, 20) = 36.96 |
| 5H  WT+HSD *vs* WT | One-way ANOVA with Tukey's multiple comparisons test | 6, 6, 6, 6 | *p* = 0.0039 | F (3, 20) = 36.96 |
| 5H  AKO+HSD *vs* WT+HSD | One-way ANOVA with Tukey's multiple comparisons test | 6, 6, 6, 6 | *p* < 0.0001 | F (3, 20) = 36.96 |
| 5J right pT231 | One-way ANOVA with Tukey's multiple comparisons test | 6, 6, 6, 6 | *p* < 0.0001 | F (3, 20) = 199.2 |
| 5J right pS262 | One-way ANOVA with Tukey's multiple comparisons test | 6, 6, 6, 6 | *p* < 0.0001 | F (3, 20) = 267.9 |
| 5J right pS396 | One-way ANOVA with Tukey's multiple comparisons test | 6, 6, 6, 6 | *p* = 0.034 | F (3, 20) = 84.25 |
| 5J right AT8 | One-way ANOVA with Tukey's multiple comparisons test | 6, 6, 6, 6 | *p* = 0.008 | F (3, 20) = 98.65 |
| 5N | One-way ANOVA with Tukey's multiple comparisons test | 6, 6, 6, 6 | *p* < 0.0001 | F (3, 20) = 188.4 |
| 6E  HSD *vs* Ctrl | One-way ANOVA with Tukey's multiple comparisons test | 6, 6, 6, 6 | *p* < 0.0001 | F (5, 30) = 43.36 |
| 6E  A1R-KO+HSD *vs* HSD | One-way ANOVA with Tukey's multiple comparisons test | 6, 6, 6, 6 | *p* = 0.0004 | F (5, 30) = 43.36 |
| 6E  Tau-KO+HSD *vs* HSD | One-way ANOVA with Tukey's multiple comparisons test | 6, 6, 6, 6 | *p* = 0.0024 | F (5, 30) = 43.36 |
| 6F  HSD *vs* Ctrl | One-way ANOVA with Tukey's multiple comparisons test | 6, 6, 6, 6 | *p* < 0.0001 | F (5, 30) = 43.09 |
| 6F  A1R-KO+HSD *vs* HSD | One-way ANOVA with Tukey's multiple comparisons test | 6, 6, 6, 6 | *p* = 0.0001 | F (5, 30) = 43.09 |
| 6F  Tau-KO+HSD *vs* HSD | One-way ANOVA with Tukey's multiple comparisons test | 6, 6, 6, 6 | *p* < 0.0001 | F (5, 30) = 43.09 |
| 6G  HSD *vs* Ctrl | One-way ANOVA with Tukey's multiple comparisons test | 6, 6, 6, 6 | *p* = 0.0013 | F (5, 30) = 11.58 |
| 6G  A1R-KO+HSD *vs* HSD | One-way ANOVA with Tukey's multiple comparisons test | 6, 6, 6, 6 | *p* = 0.0194 | F (5, 30) = 11.58 |
| 6G  Tau-KO+HSD *vs* HSD | One-way ANOVA with Tukey's multiple comparisons test | 6, 6, 6, 6 | *p* = 0.0014 | F (5, 30) = 11.58 |
| 6H  HSD *vs* Ctrl | One-way ANOVA with Tukey's multiple comparisons test | 6, 6, 6, 6 | *p* = 0.0006 | F (5, 30) = 18.13 |
| 6H  A1R-KO+HSD *vs* HSD | One-way ANOVA with Tukey's multiple comparisons test | 6, 6, 6, 6 | *p* = 0.0008 | F (5, 30) = 18.13 |
| 6H  Tau-KO+HSD *vs* HSD | One-way ANOVA with Tukey's multiple comparisons test | 6, 6, 6, 6 | *p* = 0.0062 | F (5, 30) = 18.13 |
| 6I  HSD *vs* Ctrl | One-way ANOVA with Tukey's multiple comparisons test | 6, 6, 6, 6 | *p* = 0.0002 | F (5, 30) = 17.14 |
| 6I  A1R-KO+HSD *vs* HSD | One-way ANOVA with Tukey's multiple comparisons test | 6, 6, 6, 6 | *p* < 0.0001 | F (5, 30) = 17.14 |
| 6I  Tau-KO+HSD *vs* HSD | One-way ANOVA with Tukey's multiple comparisons test | 6, 6, 6, 6 | *p* = 0.0009 | F (5, 30) = 17.14 |
| 6J  HSD *vs* Ctrl | One-way ANOVA with Tukey's multiple comparisons test | 6, 6, 6, 6 | *p* < 0.0001 | F (5, 30) = 33.82 |
| 6J  A1R-KO+HSD *vs* HSD | One-way ANOVA with Tukey's multiple comparisons test | 6, 6, 6, 6 | *p* = 0.0027 | F (5, 30) = 33.82 |
| 6J  Tau-KO+HSD *vs* HSD | One-way ANOVA with Tukey's multiple comparisons test | 6, 6, 6, 6 | *p* = 0.0425 | F (5, 30) = 33.82 |
| 6K  HSD *vs* Ctrl | One-way ANOVA with Tukey's multiple comparisons test | 6, 6, 6, 6 | *p* < 0.0001 | F (5, 30) = 45.43 |
| 6K  A1R-KO+HSD *vs* HSD | One-way ANOVA with Tukey's multiple comparisons test | 6, 6, 6, 6 | *p* < 0.0001 | F (5, 30) = 45.43 |
| 6K  Tau-KO+HSD *vs* HSD | One-way ANOVA with Tukey's multiple comparisons test | 6, 6, 6, 6 | *p* = 0.0045 | F (5, 30) = 45.43 |
| 6L  HSD *vs* Ctrl | One-way ANOVA with Tukey's multiple comparisons test | 6, 6, 6, 6 | *p* < 0.0001 | F (5, 30) = 58.26 |
| 6L  A1R-KO+HSD *vs* HSD | One-way ANOVA with Tukey's multiple comparisons test | 6, 6, 6, 6 | *p* = 0.0013 | F (5, 30) = 58.26 |
| 6L  Tau-KO+HSD *vs* HSD | One-way ANOVA with Tukey's multiple comparisons test | 6, 6, 6, 6 | *p* = 0.0029 | F (5, 30) = 58.26 |
| 7C lower | Two-tailed t test | 6, 6 | *p* < 0.0001 | t=11.00, df=10 |
| 7H left | One-way ANOVA with Tukey's multiple comparisons test | 6, 6, 6, 6 | *p* = 0.0230 | F (3, 20) = 11.67 |
| 7H middle | One-way ANOVA with Tukey's multiple comparisons test | 6, 6, 6, 6 | *p* = 0.0075 | F (3, 20) = 11.08 |
| 7H right | One-way ANOVA with Tukey's multiple comparisons test | 6, 6, 6, 6 | *p* = 0.0003 | F (3, 20) = 41.16 |
| 7L lower | Two-tailed t test | 6, 6 | *p* < 0.0001 | t=7.163, df=10 |
| 7H left | One-way ANOVA with Tukey's multiple comparisons test | 6, 6, 6, 6 | *p* = 0.0004 | F (3, 20) = 11.40 |
| 7H middle | One-way ANOVA with Tukey's multiple comparisons test | 6, 6, 6, 6 | *p* < 0.0001 | F (3, 20) = 16.94 |
| 7H right | One-way ANOVA with Tukey's multiple comparisons test | 6, 6, 6, 6 | *p* = 0.0006 | F (3, 20) = 34.90 |
| S1C | Two-tailed t test | 8, 8 | *p* = 0.0003 | t=5.512, df=14 |
| S1D | Two-way ANOVA with Tukey's multiple comparisons test | 8,8,8,8 | *p*=0.0092 Interaction  *p*<0.0001 Day  *p*<0.0001 Group | F (6, 98) = 4.157  F (6, 98) = 25.73  F (1, 98) = 37.69 |
| S1G | Two-tailed t test | 8, 8 | *p >* 0.9999 | t=0.000, df=14 |
| S1H | Two-tailed t test | 8, 8 | *p* = 0.3436 | t=0.9802, df=14 |
| S1I | Two-tailed t test | 8, 8 | *p* = 0.5972 | t=0.6459, df=14 |
| S1K | Two-tailed t test | 25, 25 | *p* < 0.0001 | t=5.535, df=48 |
| S1L | Two-tailed t test | 25, 25 | *p* < 0.0001 | t=11.46, df=48 |
| S1M | Two-tailed t test | 25, 25 | *p* < 0.0001 | t=4.536, df=48 |
| S1N | Two-tailed t test | 25, 25 | *p* < 0.0001 | t=9.075, df=48 |
| S1O | Two-tailed t test | 25, 25 | *p* = 0.0009 | t=3.536, df=48 |
| S1Q | Two-tailed t test | 17, 17 | *p* = 0.0003 | t=4.058, df=32 |
| S1R | Two-tailed t test | 17, 17 | *p* = 0.0012 | t=3.546, df=32 |
| S3B p-p65/p65 | Two-tailed t test | 6, 6 | *p* < 0.0001 | t=21.78, df=10 |
| S3B p-Stat3/Stat3 | Two-tailed t test | 6, 6 | *p* < 0.0001 | t=7.118, df=10 |
| S3D | Two-tailed t test | 6, 6 | *p* < 0.0001 | t=13.22, df=10 |
| S3E | Two-tailed t test | 6, 6 | *p* < 0.0001 | t=9.189, df=10 |
| S3F | Two-tailed t test | 6, 6 | *p* < 0.0001 | t=6.254, df=10 |
| S5C Veh | Two-tailed t test | 8, 8 | *p* = 0.0537 | t=2.354, df=14 |
| S5C 20mM 3d | Two-tailed t test | 8, 8 | *p* < 0.0001 | t=7.986, df=14 |
| S5C 20mM 7d | Two-tailed t test | 8, 8 | *p* < 0.0001 | t=17.61, df=14 |
| S6B pT231 | Two-tailed t test | 6, 6 | *p* < 0.0001 | t=7.439, df=10 |
| S6B pS262 | Two-tailed t test | 6, 6 | *p* = 0.0002 | t=5.851, df=10 |
| S6B pS396 | Two-tailed t test | 6, 6 | *p* < 0.0001 | t=8.801, df=10 |
| S6B AT8 | Two-tailed t test | 6, 6 | *p* = 0.002 | t=5.644, df=10 |
| S6B Tau5 | Two-tailed t test | 6, 6 | *p* = 0.3004 | t=1.092, df=10 |
| S7F | Two-tailed t test | 6, 6 | *p* < 0.0001 | t=18.48, df=10 |
| S7J | Two-tailed t test | 6, 6 | *p* = 0.0001 | t=6.182, df=10 |
| S7K | Two-tailed t test | 6, 6 | *p* < 0.0001 | t=8.857, df=10 |
| S7L | Two-tailed t test | 6, 6 | *p* < 0.0001 | t=6.602, df=10 |
| S8B lower | Two-tailed t test | 6, 6 | *p* < 0.0001 | t=11.15, df=10 |
| S8G left | One-way ANOVA with Tukey's multiple comparisons test | 6, 6, 6, 6 | *p* = 0.0001 | F (3, 20) = 18.51 |
| S8G middle | One-way ANOVA with Tukey's multiple comparisons test | 6, 6, 6, 6 | *p* = 0.0003 | F (3, 20) = 33.74 |
| S8G right | One-way ANOVA with Tukey's multiple comparisons test | 6, 6, 6, 6 | *p* = 0.0001 | F (3, 20) = 21.03 |
| S8H right  p-p65/p65 | One-way ANOVA with Tukey's multiple comparisons test | 6, 6, 6, 6 | *p* = 0.0002 | F (3, 20) = 27.30 |
| S8H right p-Stat3/Stat3 | One-way ANOVA with Tukey's multiple comparisons test | 6, 6, 6, 6 | *p* = 0.0001 | F (3, 20) = 22.54 |
| S8J right  pT231 | One-way ANOVA with Tukey's multiple comparisons test | 6, 6, 6, 6 | *p* < 0.0001 | F (3, 20) = 985.9 |
| S8J right  pS262 | One-way ANOVA with Tukey's multiple comparisons test | 6, 6, 6, 6 | *p* < 0.0001 | F (3, 20) = 308.2 |
| S8J right  pS396 | One-way ANOVA with Tukey's multiple comparisons test | 6, 6, 6, 6 | *p* < 0.0001 | F (3, 20) = 489.3 |
| S8J right  AT8 | One-way ANOVA with Tukey's multiple comparisons test | 6, 6, 6, 6 | *p* = 0.0064 | F (3, 20) = 87.15 |
| S9B | Two-tailed t test | 6, 6 | *p* < 0.0001 | t=12.23, df=10 |
| S9C GFAP | Two-tailed t test | 6, 6 | *p* < 0.0001 | t=10.84, df=10 |
| S9C Vimetin | Two-tailed t test | 6, 6 | *p* < 0.0001 | t=15.40, df=10 |
| S9C Clu | Two-tailed t test | 6, 6 | *p* < 0.0001 | t=6.531, df=10 |
| S9C Igfbp5 | Two-tailed t test | 6, 6 | *p* = 0.3012 | t=1.090, df=10 |
| S9C Cd63 | Two-tailed t test | 6, 6 | *p* = 0.0010 | t=4.567, df=10 |
| S9C Fxyd1 | Two-tailed t test | 6, 6 | *p* = 0.5567 | t=0.6081, df=10 |
| S9C Ctsb | Two-tailed t test | 6, 6 | *p* = 0.0008 | t=4.698, df=10 |
| S9C Cd9 | Two-tailed t test | 6, 6 | *p* = 0.0017 | t=4.235, df=10 |
| S9C Cpe | Two-tailed t test | 6, 6 | *p* = 0.6367 | t=0.4871, df=10 |
| S9C APOE | Two-tailed t test | 6, 6 | *p* = 0.0004 | t=5.191, df=10 |
| S9C Ckb | Two-tailed t test | 6, 6 | *p* = 0.8125 | t=0.2435, df=10 |
| S9C Mt2 | Two-tailed t test | 6, 6 | *p* = 0.2552 | t=1.207, df=10 |
| S9E | Two-tailed t test | 6, 6 | *p* < 0.0001 | t=8.535, df=10 |
| S9F | Two-tailed t test | 6, 6 | *p* < 0.0001 | t=9.476, df=10 |
| S9K GFAP | Two-tailed t test | 6, 6 | *p* < 0.0001 | t=12.34, df=10 |
| S9K Vimetin | Two-tailed t test | 6, 6 | *p* < 0.0001 | t=13.40, df=10 |
| S9K Clu | Two-tailed t test | 6, 6 | *p* = 0.0013 | t=4.424, df=10 |
| S9K Igfbp5 | Two-tailed t test | 6, 6 | *p* = 0.4638 | t=0.7617, df=10 |
| S9K Cd63 | Two-tailed t test | 6, 6 | *p* = 0.0384 | t=2.384, df=10 |
| S9K Fxyd1 | Two-tailed t test | 6, 6 | *p* = 0.7228 | t=0.3649, df=10 |
| S9K Ctsb | Two-tailed t test | 6, 6 | *p* = 0.0334 | t=2.465, df=10 |
| S9K Cd9 | Two-tailed t test | 6, 6 | *p* = 0.7228 | t=0.3649, df=10 |
| S9K Cpe | Two-tailed t test | 6, 6 | *p* = 0.7360 | t=0.3467, df=10 |
| S9K APOE | Two-tailed t test | 6, 6 | *p* = 0.0017 | t=4.239, df=10 |
| S9K Ckb | Two-tailed t test | 6, 6 | *p* = 0.7360 | t=0.3467, df=10 |
| S9K Mt2 | Two-tailed t test | 6, 6 | *p* = 0.7228 | t=0.3649, df=10 |

**Supplementary data Full gels for all the western blots in this study.**

**
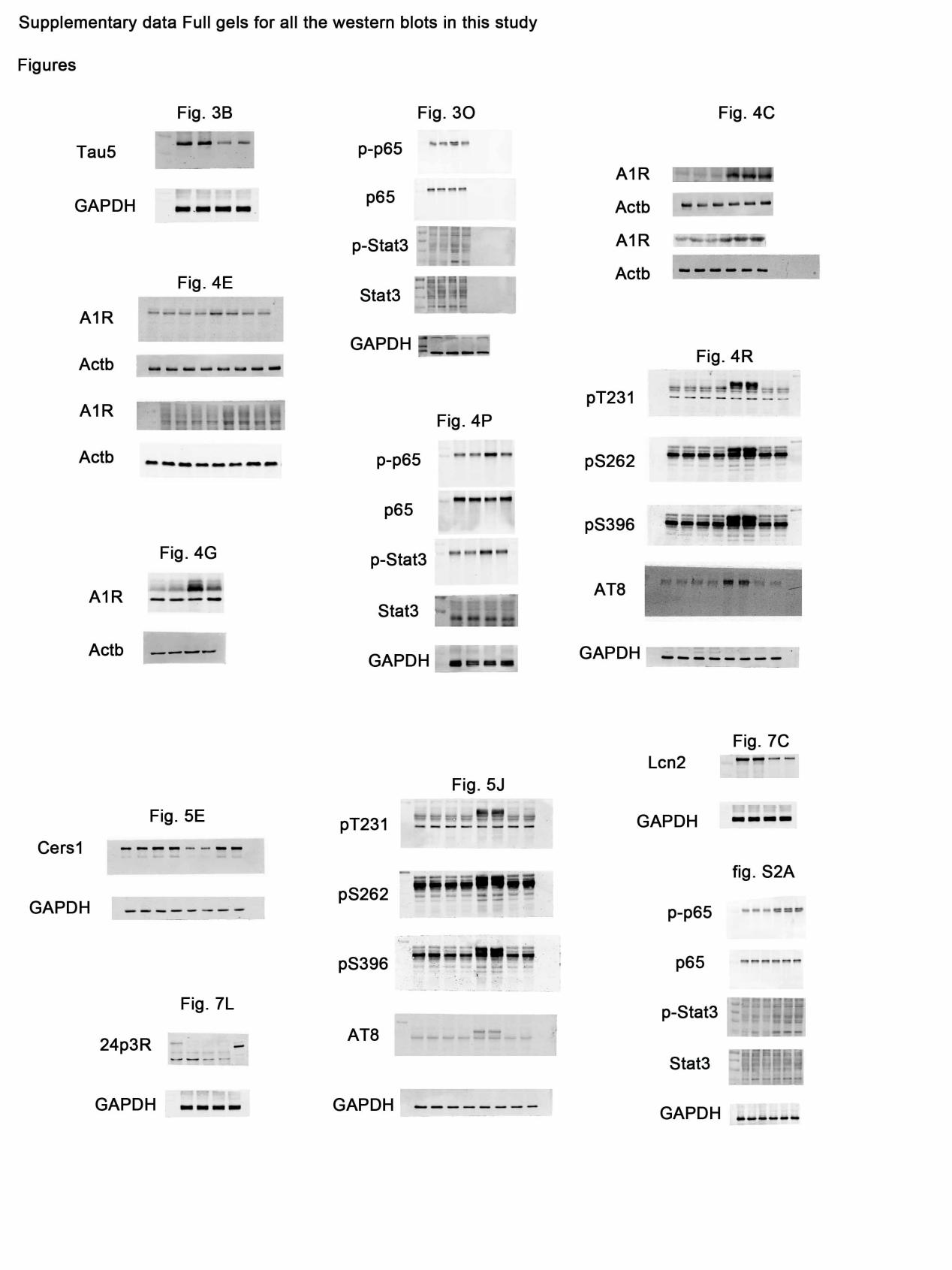
**

**
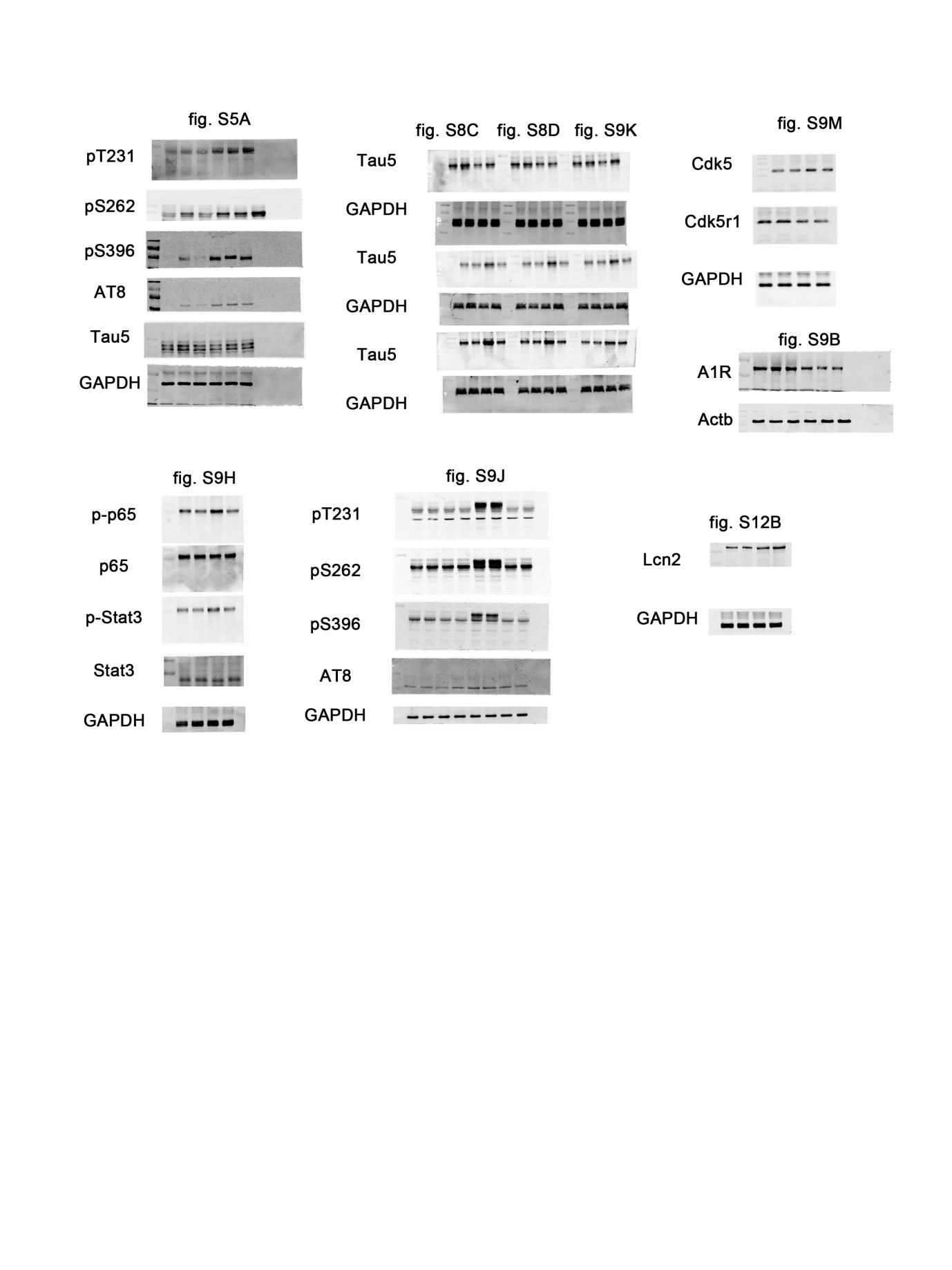
**
